# Screening embryos for polygenic disease risk: a review of epidemiological, clinical, and ethical considerations

**DOI:** 10.1093/humupd/dmae012

**Published:** 2024-05-28

**Authors:** Antonio Capalbo, Guido de Wert, Heidi Mertes, Liraz Klausner, Edith Coonen, Francesca Spinella, Hilde Van de Velde, Stephane Viville, Karen Sermon, Nathalie Vermeulen, Todd Lencz, Shai Carmi

**Affiliations:** Juno Genetics, Department of Reproductive Genetics, Rome, Italy; Center for Advanced Studies and Technology (CAST), Department of Medical Genetics, “G. d’Annunzio” University of Chieti-Pescara, Chieti, Italy; Department of Health, Ethics & Society, CAPHRI-School for Public Health and Primary Care and GROW School for Oncology and Reproduction, Maastricht University, Maastricht, The Netherlands; Department of Philosophy and Moral Sciences, Ghent University, Ghent, Belgium; Department of Public Health and Primary Care, Ghent University, Ghent, Belgium; Braun School of Public Health and Community Medicine, The Hebrew University of Jerusalem, Jerusalem, Israel; Departments of Clinical Genetics and Reproductive Medicine, Maastricht University Medical Centre, Maastricht, The Netherlands; School for Oncology and Developmental Biology, GROW, Maastricht University, Maastricht, The Netherlands; Eurofins GENOMA Group Srl, Molecular Genetics Laboratories, Department of Scientific Communication, Rome, Italy; Research Group Genetics Reproduction and Development (GRAD), Vrije Universiteit Brussel, Brussel, Belgium; Brussels IVF, UZ Brussel, Brussel, Belgium; Laboratoire de Génétique Médicale LGM, Institut de Génétique Médicale d’Alsace IGMA, INSERM UMR 1112, Université de Strasbourg, France; Laboratoire de Diagnostic Génétique, Unité de Génétique de l’infertilité (UF3472), Hôpitaux Universitaires de Strasbourg, Strasbourg, France; Research Group Genetics Reproduction and Development (GRAD), Vrije Universiteit Brussel, Brussel, Belgium; ESHRE Central Office, Strombeek-Bever, Belgium; Institute of Behavioral Science, Feinstein Institutes for Medical Research, Manhasset, NY, USA; Departments of Psychiatry and Molecular Medicine, Zucker School of Medicine at Hofstra/Northwell, Hempstead, NY 11549, USA; Braun School of Public Health and Community Medicine, The Hebrew University of Jerusalem, Jerusalem, Israel

**Keywords:** preimplantation genetic testing, polygenic risk scores, polygenic embryo screening, PGT-P, statistical genetics, disease risk reduction, ethical considerations, clinical considerations

## Abstract

**BACKGROUND:**

The genetic composition of embryos generated by *in vitro* fertilization (IVF) can be examined with preimplantation genetic testing (PGT). Until recently, PGT was limited to detecting single-gene, high-risk pathogenic variants, large structural variants, and aneuploidy. Recent advances have made genome-wide genotyping of IVF embryos feasible and affordable, raising the possibility of screening embryos for their risk of polygenic diseases such as breast cancer, hypertension, diabetes, or schizophrenia. Despite a heated debate around this new technology, called polygenic embryo screening (PES; also PGT-P), it is already available to IVF patients in some countries. Several articles have studied epidemiological, clinical, and ethical perspectives on PES; however, a comprehensive, principled review of this emerging field is missing.

**OBJECTIVE AND RATIONALE:**

This review has four main goals. First, given the interdisciplinary nature of PES studies, we aim to provide a self-contained educational background about PES to reproductive specialists interested in the subject. Second, we provide a comprehensive and critical review of arguments for and against the introduction of PES, crystallizing and prioritizing the key issues. We also cover the attitudes of IVF patients, clinicians, and the public towards PES. Third, we distinguish between possible future groups of PES patients, highlighting the benefits and harms pertaining to each group. Finally, our review, which is supported by ESHRE, is intended to aid healthcare professionals and policymakers in decision-making regarding whether to introduce PES in the clinic, and if so, how, and to whom.

**SEARCH METHODS:**

We searched for PubMed-indexed articles published between 1/1/2003 and 1/3/2024 using the terms ‘polygenic embryo screening’, ‘polygenic preimplantation’, and ‘PGT-P’. We limited the review to primary research papers in English whose main focus was PES for medical conditions. We also included papers that did not appear in the search but were deemed relevant.

**OUTCOMES:**

The main theoretical benefit of PES is a reduction in lifetime polygenic disease risk for children born after screening. The magnitude of the risk reduction has been predicted based on statistical modelling, simulations, and sibling pair analyses. Results based on all methods suggest that under the best-case scenario, large relative risk reductions are possible for one or more diseases. However, as these models abstract several practical limitations, the realized benefits may be smaller, particularly due to a limited number of embryos and unclear future accuracy of the risk estimates. PES may negatively impact patients and their future children, as well as society. The main personal harms are an unindicated IVF treatment, a possible reduction in IVF success rates, and patient confusion, incomplete counselling, and choice overload. The main possible societal harms include discarded embryos, an increasing demand for ‘designer babies’, overemphasis of the genetic determinants of disease, unequal access, and lower utility in people of non-European ancestries. Benefits and harms will vary across the main potential patient groups, comprising patients already requiring IVF, fertile people with a history of a severe polygenic disease, and fertile healthy people. In the United States, the attitudes of IVF patients and the public towards PES seem positive, while healthcare professionals are cautious, sceptical about clinical utility, and concerned about patient counselling.

**WIDER IMPLICATIONS:**

The theoretical potential of PES to reduce risk across multiple polygenic diseases requires further research into its benefits and harms. Given the large number of practical limitations and possible harms, particularly unnecessary IVF treatments and discarded viable embryos, PES should be offered only within a research context before further clarity is achieved regarding its balance of benefits and harms. The gap in attitudes between healthcare professionals and the public needs to be narrowed by expanding public and patient education and providing resources for informative and unbiased genetic counselling.

## Graphical Abstract

**Figure:**
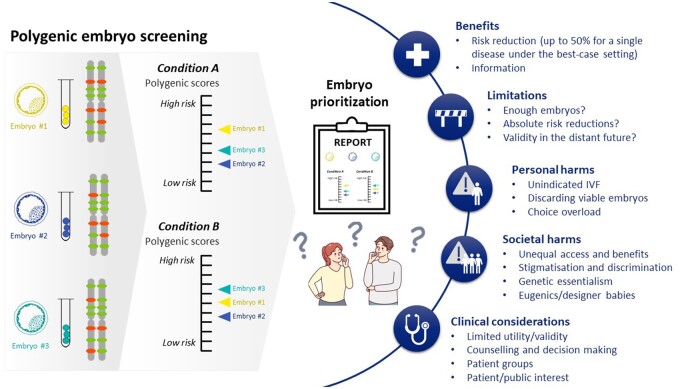
The principles, estimated benefits, personal and societal harms, and clinical considerations of prioritizing IVF embryos based on their risk for late-onset, polygenic conditions.

## Introduction: polygenic embryo screening

In preimplantation genetic testing (PGT), a biopsy is taken from a developing *in vitro* fertilization (IVF) embryo prior to its transfer to the uterus in order to test its genetic composition. In current practice, the biopsy consists of a few cells removed 5–7 days post-fertilization from the trophectoderm of embryos that have reached the blastocyst stage (Cimadomo *et al.*, 2020). PGT is currently used for testing for monogenic variants (PGT-M), large structural variants (PGT-SR), and aneuploidy (PGT-A). PGT-M is offered to patients who carry high-penetrance pathogenic variants who are at risk of giving birth to affected children (ESHRE PGT Consortium Steering Committee *et al.*, 2020). PGT-SR is indicated to patients who carry a chromosomal rearrangement and are thus at a risk of subfertility, pregnancy loss, or even congenital abnormalities in the offspring (ESHRE PGT Consortium Steering Committee *et al.*, 2020). In contrast, PGT-A is usually performed as a screening test under the premise that transferring a euploid embryo will reduce the time to pregnancy and the risk of miscarriage (Scott *et al.*, 2013; Capalbo *et al.*, 2022).

The recent reduction in the cost of genotyping and sequencing has led to the development of ‘universal’ (or ‘comprehensive’) PGT by multiple clinics worldwide. This class of methods, which can simultaneously perform all three types of PGT, involves (usually shallow) whole-genome sequencing or genome-wide genotyping of each embryo and, often, also the intended parents and additional close relatives. Algorithms then combine the embryo and family data for an accurate reconstruction of the entire genome sequence of each embryo (Thornhill *et al.*, 2015; Yan *et al.*, 2015; Zamani Esteki *et al.*, 2015; Backenroth *et al.*, 2019; Masset *et al.*, 2019; Treff *et al.*, 2019b; Davies *et al.*, 2020; Murphy *et al.*, 2020; Yuan *et al.*, 2020; Chen *et al.*, 2021; Zhang *et al.*, 2021; De Witte *et al.*, 2022; Kumar *et al.*, 2022; Masset *et al.*, 2022; Xia *et al.*, 2022; Xie *et al.*, 2022; Backenroth *et al*., 2023; Hornak *et al.*, 2023; Janssen *et al.*, 2023). This raises the prospects of screening IVF embryos for additional conditions.

**Figure 1.**
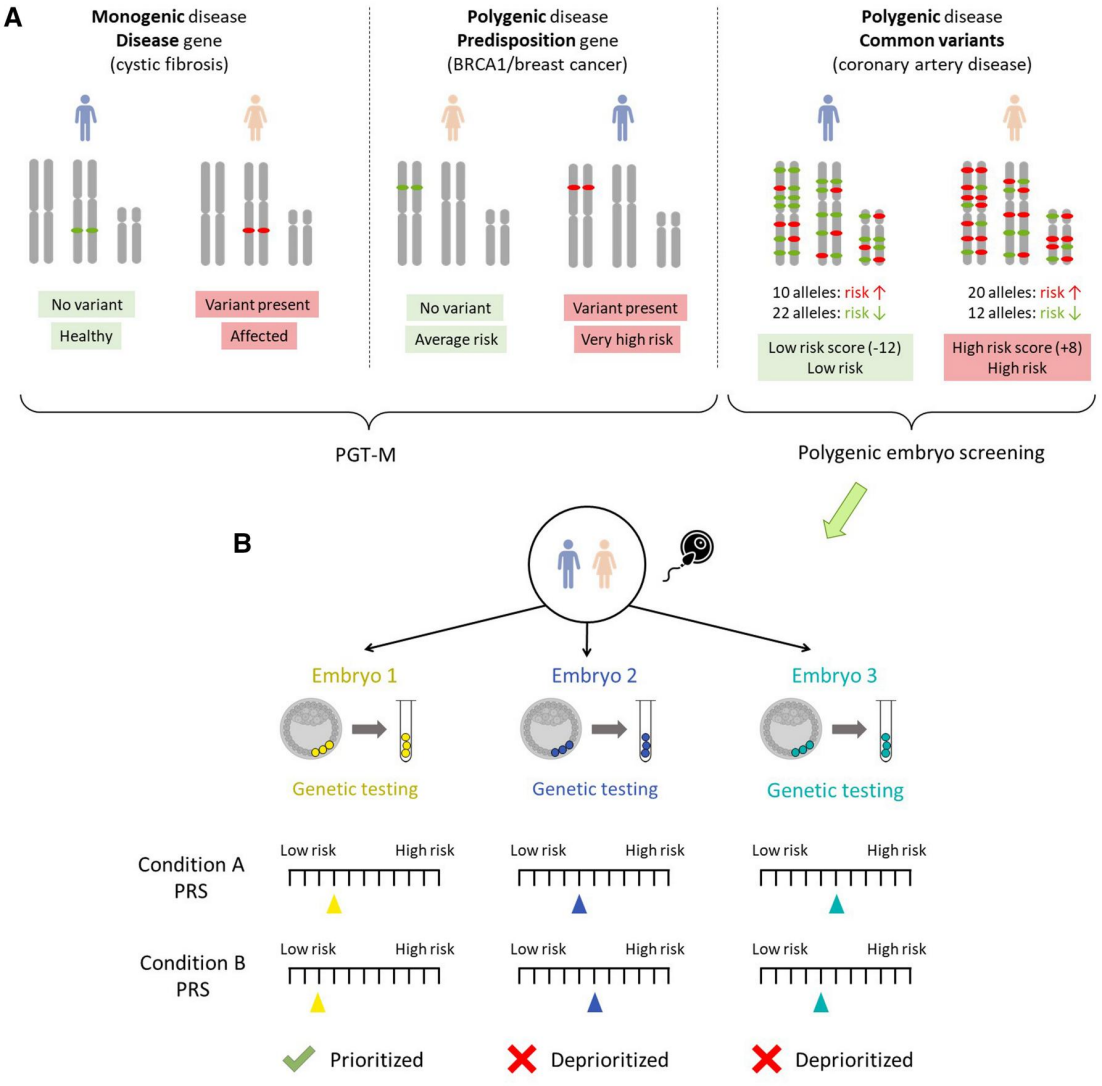
**Polygenic risk scores and polygenic embryo screening.** (**A**) Genetic testing for monogenic and polygenic diseases. For monogenic disorders (e.g. cystic fibrosis; left panel), the disease status is determined by the genotype in pathogenic variants of near-complete penetrance in the disease gene. Carriers of such variants can use PGT-M to avoid transmission to the next generation. For polygenic diseases, the risk is influenced by multiple variants throughout the genome, as well as by non-genetic factors. For some complex diseases (e.g. breast cancer; middle panel), high-penetrance variants in predisposition genes confer moderate to high risk to carriers, who can also use PGT-M to avoid transmission to future children. However, high-penetrance variants are rare, and the risk is otherwise determined by numerous common variants, each associated with a small increase or decrease in risk (right panel). The polygenic risk score (PRS) is the overall predicted risk based on the genotypes in all disease-associated common variants (here illustrated, for simplicity, as the difference between the number of risk and protective alleles). Individuals with high PRS will have higher-than-average risk, but will not necessarily become affected. (**B**) Polygenic embryo screening (PES). For each IVF embryo, DNA from a trophectoderm biopsy is sequenced/genotyped and PRSs are computed for one or more conditions. The risk profiles of the embryos are compared and a single embryo is prioritized for transfer according to a pre-determined criterion or ‘selection strategy’. In this illustration, under a strategy of prioritizing the embryo with the lowest average risk across conditions, embryo 1 would be prioritized. Alternative selection strategies may include exclusion of high-risk embryos and selection at random among the others or selection of the embryo with the lowest weighted average PRS. PES may also be performed only for informational purposes, using morphology or other factors for selection.

PGT-M was traditionally offered to avoid monogenic diseases, which are caused by the disruption of a single gene (Fig. 1A). However, most human traits and diseases with a genetic basis are polygenic, or complex, namely, they are influenced by hundreds or thousands of loci throughout the genome (in addition to numerous non-genetic factors). Polygenic traits include most anthropometric (e.g. height or BMI), cardiometabolic (e.g. cholesterol, glucose levels, or blood pressure), cognitive, behavioural, and personality traits. Polygenic diseases include most common and chronic conditions, including coronary artery disease, hypertension, stroke, type 1 and type 2 diabetes, most cancers, age-related macular degeneration, glaucoma, asthma, multiple sclerosis, inflammatory bowel disease, Parkinson’s disease, Alzheimer’s disease, schizophrenia, and major depressive disorder, to give a few examples (World Health Organization, 2021).

For many polygenic diseases, rare, high-penetrance pathogenic variants have been discovered in so-called predisposition genes. For example, carriers of pathogenic variants in *BRCA1*, *APC*, or *SCN5A* are at a higher risk of breast and ovarian cancer, colon cancer, or heart disease, respectively, compared to non-carriers (Dorling *et al.*, 2021; American College of Medical Genetics and Genomics, 2023; Miller *et al.*, 2023). The magnitude of the risk can vary considerably across predisposition genes and variants. PGT-M is now routinely used to avoid the transmission of these variants to future children (Albujja *et al.*, 2023; Spinella *et al.*, 2023) (Fig. 1A). However, most individuals affected with polygenic diseases do not carry high-penetrance pathogenic variants (Sinicrope, 2018; PDQ^®^ Cancer Genetics Editorial Board, 2023). Rather, disease risk is influenced by numerous common variants genome-wide, each of which is only weakly associated with the disease (O’Connor *et al.*, 2019; Claussnitzer *et al.*, 2020; Yengo *et al.*, 2022; Abdellaoui *et al.*, 2023). Most of these variants were unknown until about a decade ago.

In recent years, studies of polygenic diseases have been pushed forward by large biobanks and research consortia that have aggregated genomic and phenotypic data from hundreds of thousands of individuals (Bycroft *et al.*, 2018; Ishigaki *et al.*, 2020; Mars *et al.*, 2020; Aragam *et al.*, 2022; Yengo *et al.*, 2022; Kurki *et al.*, 2023; Walters *et al.*, 2023). Genome-wide association studies (GWAS) in these cohorts led to the discovery of numerous robust associations between common variants and complex diseases (Loos, 2020; Abdellaoui *et al.*, 2023). These discoveries led in turn to the development of increasingly powerful polygenic risk scores (PRS) for personalized disease risk prediction. A PRS for a given disease is a count of risk alleles carried by an individual, weighting alleles by their strength of association (Fig. 1A). Thus, a PRS provides individuals with an estimate of their genetic predisposition to a disease based on the presence or absence of common variants associated with the disease (Wray *et al.*, 2007; Lewis and Vassos, 2022; Raben *et al.*, 2022; Wang *et al.*, 2022).

Given the full embryo’s genome, it is possible to compute the embryo’s polygenic scores for any disease (or trait) of interest. These advances suggest the possibility of prioritizing embryos for transfer, or even discarding embryos, based on these scores. We call this Polygenic Embryo Screening (PES) (Lencz *et al.*, 2021). The procedure is also commonly known as PGT-P, i.e. PGT for Polygenic conditions (Treff *et al.*, 2019b), although we prefer not to use this term in order to distinguish PES from the more diagnostic forms of PGT. PES is also known as Embryo Selection based on Polygenic Scores (ESPS) (Turley *et al.*, 2021) and PRS for Embryo Selection (PRS-ES) (Polyakov *et al.*, 2022). PES is illustrated in Fig. 1B. PES could be offered regardless of the medical indication for the IVF treatment, or even the lack thereof, and it could be associated with multiple embryo prioritization strategies (Fig. 1B).

PES is currently offered, either commercially or under research protocols, by three US-based companies: Genomic Prediction (offering PES commercially under the name LifeView) (Treff *et al.*, 2019a,b), Orchid Health (Xia *et al.*, 2022, 2024) and MyOme (Kumar *et al.*, 2022). PES is marketed as a technology to reduce complex disease risk in future offspring by selecting the embryo with the best predicted health, thereby minimizing the impact of chance. However, PES could also be used just for informational purposes. PGT is unregulated in the USA, and therefore PES is permitted there. The number of couples who have undergone PES with Genomic Prediction is in the low hundreds (Genomeweb.com, 2021; Eccles *et al.*, 2022) and a few babies were reported to be born (Bloomberg News, 2021). Nevertheless, given that PES may become a means towards ‘designer babies’, it was already very widely covered by the media (https://www.polygenicembryo.org/media), including in news reports by leading scientific journals (Pagnaer *et al.*, 2021; Kamenova and Haidar, 2022; Kozlov, 2022).

In the rest of this article, we review (i) polygenic scores and their limitations; (ii) the expected clinical utility of PES under the best-case scenario; (iii) practical and technical factors that are likely to limit the expected utility; (iv) possible harms that may affect individuals using PES; (v) possible societal harms due to PES; (vi) considerations regarding clinical implementation of PES; and (vii) ethical considerations.

We focus here on PES for disease risk. The utility of PES for non-disease traits is predicted to be limited (Karavani *et al.*, 2019; Turley *et al.*, 2021), and, even in the best case, it has no clear medical benefits. Further, it involves a very different set of practical, social, and ethical concerns. It is therefore outside the scope of this review.

## Polygenic risk scores and their limitations

### Development of polygenic risk scores

A PRS is computed using GWAS summary statistics. In a GWAS, single-nucleotide polymorphisms (SNPs) are individually tested for an association with the investigated condition. In a typical GWAS, each individual is genotyped at ∼500k–1M SNPs. Millions of additional ungenotyped SNPs can be tested after imputation, in which their genotypes are predicted based on their linkage disequilibrium with genotyped SNPs in an external, deeply sequenced reference panel (Das *et al.*, 2018). The association *P*-value and effect size (e.g. log-odds ratio) of each SNP are publicly released (Sollis *et al.*, 2023). A PRS for an individual is computed as a sum over all SNPs, adding for each SNP the number of risk-increasing alleles carried by the individual, each multiplied by the effect size. As a pre-processing step, linked SNPs and SNPs with insignificant effects are removed, based on cutoffs selected to maximize prediction accuracy in an external dataset (Choi *et al.*, 2020). More advanced methods for generating a PRS take into account the effect of linkage disequilibrium on the association statistics (Vilhjalmsson *et al.*, 2015), the genetic architecture of the disease (Ge *et al.*, 2019), ancestry differences (Amariuta *et al.*, 2020), GWAS results from related traits (Chung *et al.*, 2019), and the functional annotation of the associated variants (Márquez-Luna *et al.*, 2021). PRSs have been developed for almost all chronic conditions of medical interest and for many non-pathological traits (Lambert *et al.*, 2021; Wang *et al.*, 2022).

Variants screened in PGT-M (in monogenic disease genes or in predisposition genes) differ from variants screened in PES in a number of important ways (Table 1). PGT-M variants (i) are usually rare; (ii) are usually protein coding; (iii) directly capture the relevant genetic element; (iv) have a partly or fully understood molecular mechanism; (v) can be dominant or recessive (at least approximately (Barton *et al.*, 2022; Heyne *et al.*, 2023)); and (vi) have relatively high penetrance: a heterozygous or homozygous carrier (depending on dominance) will be affected with moderate to high probability or even with certainty. In contrast, variants in polygenic scores (i) are usually common; (ii) are rarely coding; (iii) are usually not themselves the causal element, but are linked to unknown, likely regulatory, causal variants (Schaid *et al.*, 2018); (iv) typically have no clearly defined mechanism; (v) indicate an additive risk increase, with little or no dominance or interaction effects (Hivert *et al.*, 2021; Pazokitoroudi *et al.*, 2021; Palmer *et al.*, 2023); and (vi) are associated with small changes in disease risk.

**Table 1.** A comparison of the key contrasting properties of variants screened during PGT-M and variants in polygenic scores (screened in PES).

Genetic risk factor	PGT-M variants	Polygenic score variants
Variant frequency	Rare	Common
Variant characteristics	Protein-coding, causal, known mechanism	Non-coding, non-causal (linked to unknown causal variants), unknown mechanism
Principal genetic model	Dominant/recessive	Additive
Diagnostic	Usually (moderate to very high or complete penetrance)	Not (small relative risk)

PGT-M, preimplantation genetic testing for monogenic diseases; PES, polygenic embryo screening.

### Applications of polygenic risk scores

When applied in the general population, PRSs can identify individuals at a substantially increased risk for a given disease. For example, individuals in the top 5% of the PRS distribution for coronary artery disease were shown to be at a 5-fold increased risk in comparison to the population mean (Khera *et al.*, 2018; Patel *et al.*, 2023). These individuals can have a higher risk than carriers of known monogenic risk alleles for hypercholesterolemia, despite having no family history. It has been suggested that high-PRS individuals could then be subjected to tailored screening, monitoring, lifestyle modifications, or medical interventions (Mars *et al.*, 2020).

Several studies have shown that a PRS can improve risk assessment even when combined with clinical, demographic, or monogenic risk factors, including family history (Davies *et al.*, 2020; Fahed *et al.*, 2020; Gao *et al.*, 2021; Briggs *et al.*, 2022; Hassanin *et al.*, 2022; Khan *et al.*, 2022; Mars *et al.*, 2022; Ghouse *et al.*, 2023; Honigberg *et al.*, 2023). However, the improvement can be modest, and it is often insignificant, particularly at relatively older ages (Elliott *et al.*, 2020; Mosley *et al.*, 2020; Isgut *et al.*, 2021; Sun *et al.*, 2021; Koch *et al.*, 2023; Marston *et al.*, 2023; Saadatagah *et al.*, 2023; Urbut *et al.*, 2023). Nevertheless, a number of trials testing the clinical utility of PRS-based risk prediction were recently initiated, most notably for breast cancer (BOADICEA/CanRisk tool) (Lee *et al.*, 2019). A few recent reviews have examined the clinical potential of PRS screening in adults (Gladding *et al.*, 2020; Green *et al.*, 2022).

### Limitations of polygenic risk scores

Despite their promise, PRSs also have significant limitations as predictors of disease risk. First, most complex diseases are influenced not only by genetic factors but also by non-genetic risk factors (e.g. environmental, lifestyle, as well as random molecular events). Second, PRSs only incorporate common variants and usually only consider SNPs and small insertions/deletions. Rare variants, repeat expansions, structural variants, and variants in repetitive genomic regions are usually not considered. Third, even for common variants, the precision of their estimated associated risk is limited by the GWAS sample size (Dudbridge, 2013), such that PRSs account for only a small proportion of the heritability attributed to common variants (International Schizophrenia Consortium *et al.*, 2009; Wray *et al.*, 2021). Fourth, diseases are often heterogeneous and difficult to diagnose, leading to measurement errors (Wray and Maier, 2014; Cai *et al.*, 2020). The requirements from GWAS subjects to survive and to agree to participate in the study can also introduce bias (Anderson *et al.*, 2011; DudbridGe *et al.*, 2019; Pirastu *et al.*, 2021; Tyrrell *et al.*, 2021; Mignogna *et al.*, 2023; Schoeler *et al.*, 2023). Fifth, PRSs often capture effects that are ‘indirect’, which only predict differences between individuals from different families. Alleles with indirect effects can be, for example, correlated with population affiliation or with the parental-provided environment (Selzam *et al.*, 2019; Young *et al.*, 2019; Morris *et al.*, 2020; Howe *et al.*, 2022) and would therefore be ineffective in predicting differences between sibling embryos. Sixth, given that most GWASs were conducted in populations of European genetic ancestries (Duncan *et al.*, 2019; Mills and Rahal, 2020), PRSs are less accurate in people of other ancestries, particularly African (Martin *et al.*, 2017; Fahed *et al.*, 2021; Mars *et al.*, 2022; Prive *et al.*, 2022; Ding *et al.*, 2023). Finally, even for people of the same ancestry, PRS accuracy can vary with age, sex, country, and socioeconomic status (Mostafavi *et al.*, 2020; Jermy *et al.*, 2023; Wang *et al.*, 2023).

Importantly, the PRS of individuals does not determine whether they will become affected, but it can only assign them to different levels of risk. Thus, even individuals assigned as high-risk can remain healthy. For example, suppose a PRS identifies individuals having three times higher risk compared to the population average. Then, even for a relatively common disease of prevalence of 10%, only 30% of the high-risk individuals would become affected. On the other hand, the majority of those affected will be classified by their PRS as low-risk, regardless of the specific cutoff applied (Wald and Old, 2019; Sud *et al.*, 2021). Thus, given their both low positive predictive value and low sensitivity, PRSs are not intended to serve as diagnostic tests. Rather, a PRS is a probabilistic estimate of disease risk.

Whether PES would lead, despite the above-mentioned limitations, to reduced disease risk in children is the subject of the next section.

## The expected benefits of polygenic embryo screening

**Figure 2.**
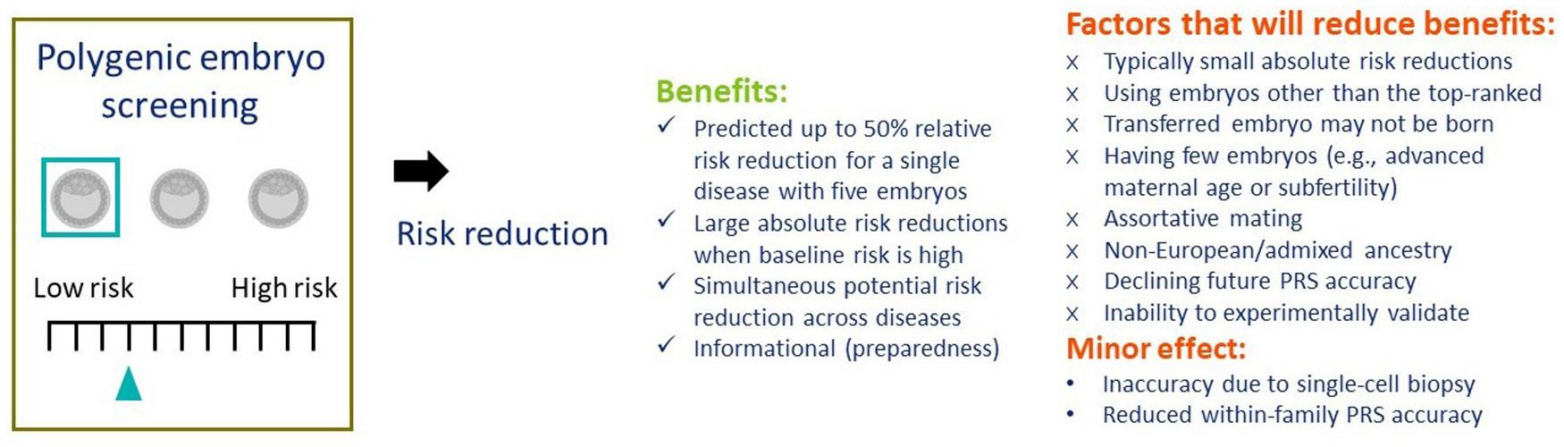
**A visual summary of the potential benefits of polygenic embryo screening along with practical limitations that will make risk reductions smaller.** PRS, polygenic risk score.

The main theoretical benefit of PES is the prospect of reducing disease risk in future children. In this section, we describe estimates for the expected risk reduction that have been obtained using various approaches. PES may also have ‘informational’ benefits to parents and children. However, given the lack of data on the value of such benefits, we only briefly review them later. See Fig. 2 for a summary of the expected benefits and the main limiting factors.

### Risk reductions under the best-case scenario

Published risk reduction estimates assume a single oocyte retrieval cycle and that PRSs for one or more diseases are computed for each embryo. Then, a single embryo is selected for transfer based on having the most desired (lowest) PRS or combination of PRSs. The risk reduction is estimated by comparing the predicted disease risk of children born after embryo screening to the risk in the general population. In the next subsection, we describe modelling and simulation approaches that were used to estimate the degree of risk reduction. We then survey risk reduction estimates derived under idealized modelling assumptions and discuss various practical factors that may diminish the achieved risk reduction.

#### How is the risk reduction estimated?

**Figure 3.**
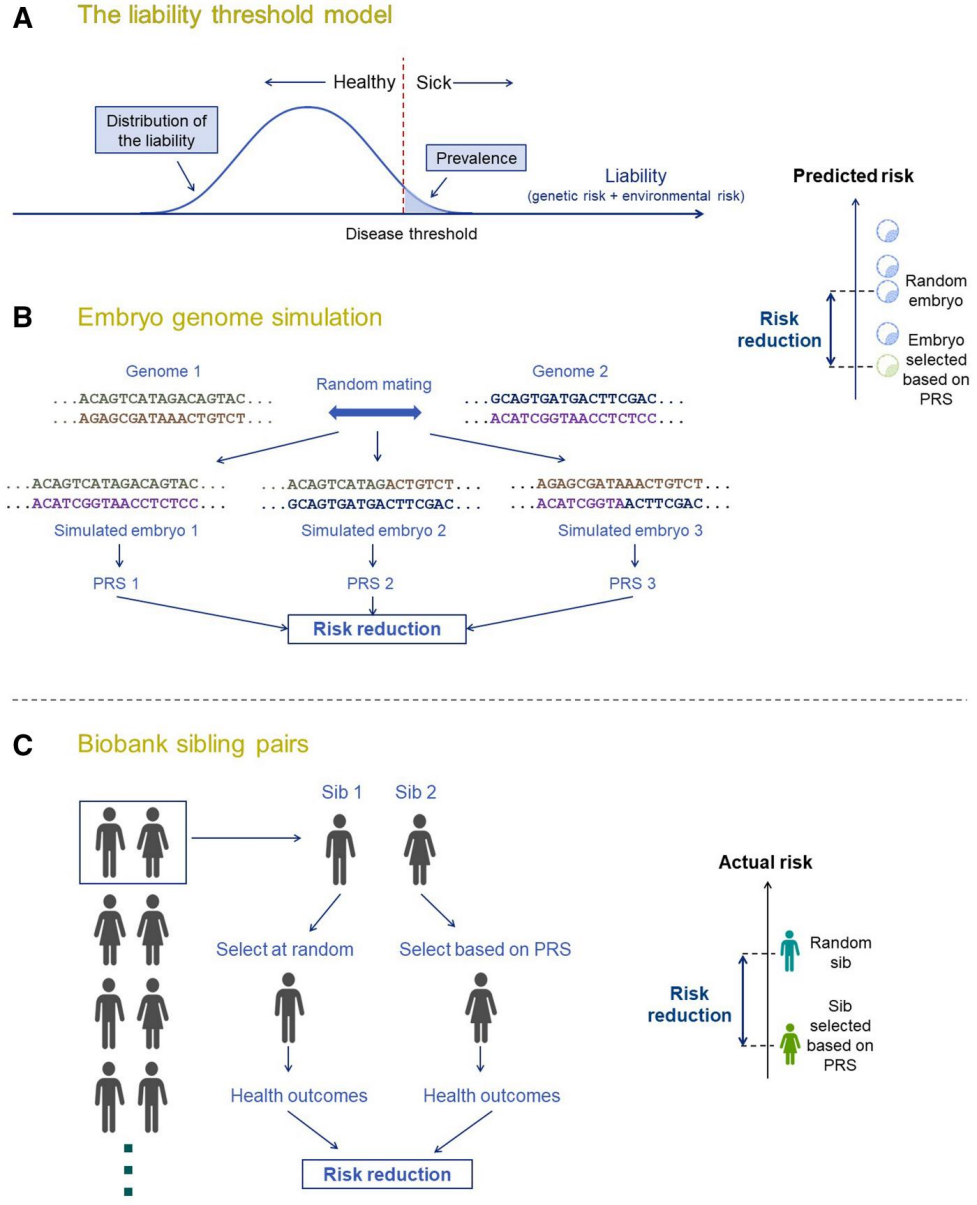
**Three approaches for predicting the risk reduction due to polygenic embryo screening**. (**A**) Statistical modelling using the liability threshold model. In this model, a disease is assumed to have an underlying (normally distributed) continuous liability, representing the sum of genetic and non-genetic risk factors. Individuals are affected if their liability is above a threshold. The liabilities of sibling embryos can be modelled based on quantitative genetic theory, and the risk of an embryo selected based on its polygenic risk score (PRS) can be predicted. The risk reduction is defined as the difference in risk between children born without selection and children born after being selected based on one or more PRSs (diagram on the right). (**B**) *Simulations based on real genomes*. In this approach, the genomes of real (unrelated) individuals are randomly mated in silico. For each ‘virtual couple’, genomes of embryos are simulated based on Mendel’s laws and realistic recombination rates. A PRS is computed for each embryo and the risk of each embryo is predicted. The risk reduction is then computed as in (A). (**C**) *Simulations based on sibling pairs*. In this approach, pairs (or larger sets) of siblings are identified in large cohorts such as the UK Biobank. For each pair of siblings, one or more PRSs are computed, and a sibling is selected either at random or based on having a favourable PRS profile. Real health outcomes are then extracted from the biobank, and the risk reduction is computed as the difference in the proportion of affected siblings between selection based on PRS and random selection (diagram at the bottom right).

Three approaches have been proposed to estimate the risk reduction due to PES: one based on statistical modelling and two based on simulations (Fig. 3). All methods require a ‘selection strategy’ under which an embryo is selected for transfer. When screening for a single disease, it is assumed that the embryo with the lowest PRS is selected. See the end of this subsection for considerations when screening for multiple diseases.

The first approach is strictly statistical, and is based on the ‘liability threshold model’: a simple and powerful framework to link genetic predisposition to disease risk (Fig. 3A). Under the model, complex diseases, which are generally considered dichotomous (affected/unaffected), have an underlying, unobserved, continuous liability, representing the sum of numerous genetic and non-genetic risk factors. Individuals are affected if their liability exceeds a threshold. Using probability theory, researchers can calculate the probability of being affected for either a random embryo (i.e. without PES) or the embryo with the lowest PRS for a given disease. The risk reduction is then defined as the difference in risk between children born with or without PES (Gwern net., 2016; Treff *et al.*, 2019a; Lencz *et al.*, 2021; Turley *et al.*, 2021; ORCHID GUIDES, 2023) (Fig. 3A).

The other two approaches involve simulations. In the first, real genomes of unrelated individuals are used to create ‘virtual couples’, and then the genomes of two or more sibling embryos are simulated (Lencz *et al.*, 2021). A PRS is computed for each simulated embryo and the risk is compared between hypothetical IVF cycles with and without PES (Fig. 3B).

The second simulation approach is based on real genomes and real phenotypic data for sets of two or more siblings (Treff *et al.*, 2019a, 2020a; ORCHID GUIDES, 2023). In this approach, a sibling is selected from each sibship either at random or based on its PRS. The proportion of selected siblings who are affected is then compared between the two selection strategies (Fig. 3C). This approach makes the smallest number of assumptions, but it is limited by the availability of genomic and phenotypic data.

All three approaches (Fig. 3A–C) assume that all embryos tested are viable and will be born following transfer. See more discussion of this issue in the section on *Factors that will limit the expected gains*.

Embryo selection based on the risk of multiple diseases raises a few additional considerations. First, as the number of diseases increases, it becomes increasingly unlikely that a single embryo would have the lowest PRS for all diseases. In some cases, all embryos will have a high risk (e.g. top few PRS percentiles) for at least one disease (https://gist.github.com/scarmi/3835f1ca0a9c77d61dbdcf8d85c91daf). Second, various selection criteria may be proposed, such as selecting the embryo with the lowest average PRS, the lowest weighted average PRS (based on the assumed harm of each disease), or the lowest probability of having *any* disease. Third, initial modelling results (Gwern net, 2016; Karavani *et al.*, 2019) suggest that with more diseases screened, the risk reduction per-disease will diminish; however, the overall health gains are expected to increase.

#### Published risk reduction estimates

Recent papers modelling PES outcomes for polygenic disorders have consistently found that relative risk reduction could be substantial (Treff *et al.*, 2019a, 2020a; Lencz *et al.*, 2021; Turley *et al.*, 2021). These results are contrary to models of PES for quantitative traits, such as height or intelligence, where the expected gains are small and uncertain (Karavani *et al.*, 2019; Turley *et al.*, 2021). Intuitively, the large relative risk reductions for diseases could be explained by the dichotomous nature of the disease state (Fig. 3A): even if the reduction in the (continuous) genetic liability is small, it may still be sufficient in some cases to avoid disease (Lencz *et al.*, 2021).

Some authors have argued that given that IVF embryos are genetic siblings, their PRSs would be too similar for any significant risk reductions to be achievable (Forzano *et al.*, 2022; Polyakov *et al.*, 2022). However, both classical modelling (Wray *et al.*, 2019) and recent empirical results (Lencz *et al.*, 2021; Lello *et al.*, 2023) suggest that the PRSs of sibling embryos could be much more different than what might be intuitively expected. Specifically, the variance in a PRS across embryos is expected to be as high as half the variance observed across the entire population (see p. 1137 in Wray *et al.*, 2019 for an intuitive derivation).

Specific estimates for relative risk reductions when screening for a single disease have varied across the available literature. At the top end, a study by Genomic Prediction used the sibling approach (Fig. 3C) to evaluate the relative risk reduction when screening for type 1 diabetes in a high-risk cohort (Treff *et al.*, 2019a). They found that selecting the sibling with the lowest PRS would have reduced the risk by 45% for two siblings and 72% for five. On the other hand, lower estimates were obtained by Turley *et al.*, who used the liability threshold model (Fig. 3A) to evaluate the relative risk reduction across each of nine disorders: type 1 and type 2 diabetes, breast, prostate, and testicular cancer, melanoma, coronary artery disease, hypercholesterolemia, and hypertension (Turley *et al.*, 2021). They found relative risk reductions between 15% and 35% (median: 22%) for couples of European ancestries who selected the embryo with the lowest PRS out of ten embryos. The predicted risk reduction for couples of non-European ancestries (particularly African) was substantially lower.

**Figure 4.**
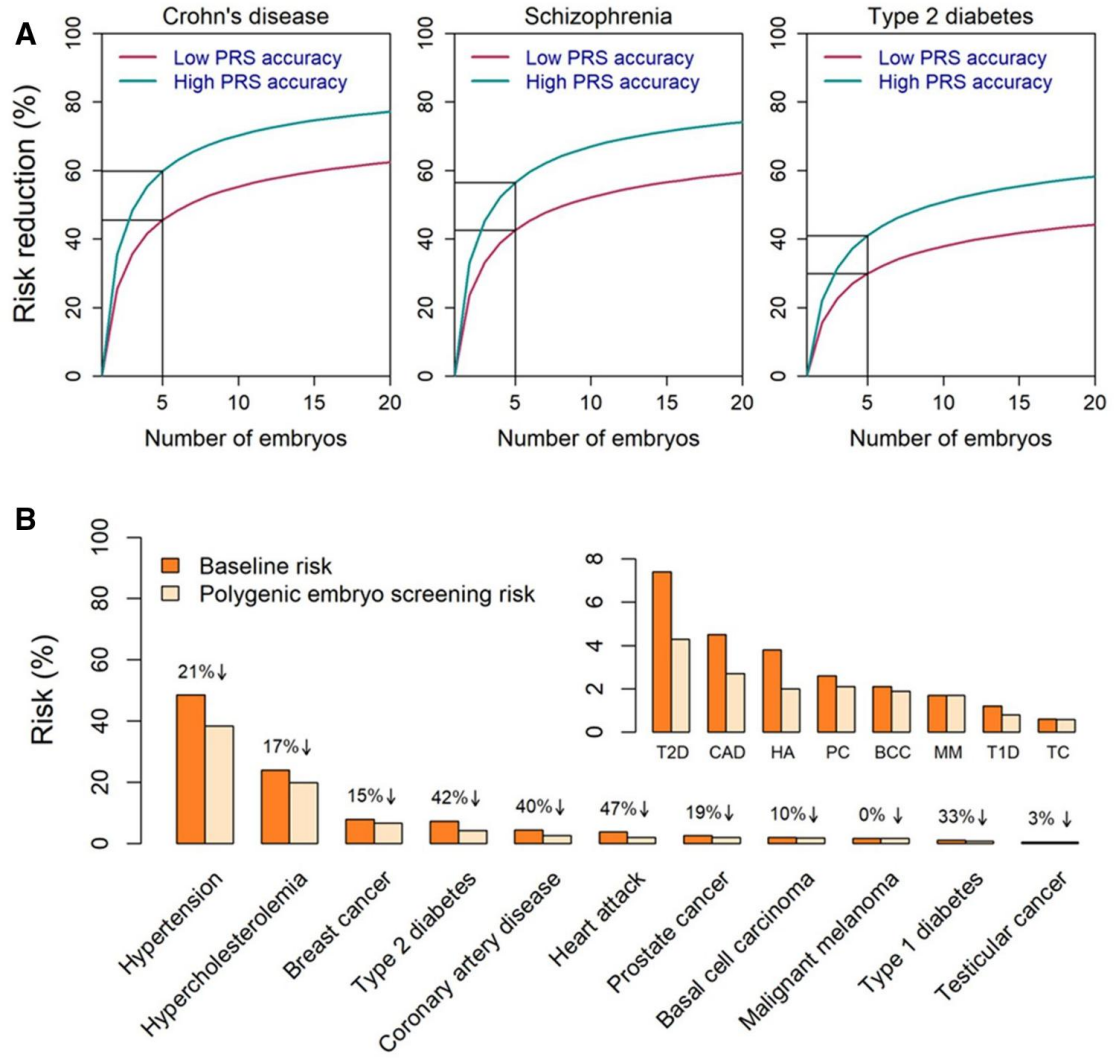
**The predicted risk reduction with polygenic embryo screening (PES).** (**A**) The predicted relative risk reduction versus the number of embryos when selecting embryos based on their polygenic risk score (PRS) for a single disease. The figure is based on a statistical modelling approach (Lencz *et al.*, 2021) (Fig. 3A). We assume that embryos from a single oocyte retrieval cycle were screened with a PRS for a single disease and that the embryo with the lowest PRS was selected for transfer and was born. For three representative diseases, the figure shows the predicted relative risk reduction versus the number of embryos. Risk reduction curves are shown for two values of PRS accuracy, corresponding to lower and upper bounds on the current performance of PRS-based risk prediction for these diseases. For additional technical details, see the caption of Fig. 5. (**B**) The predicted risk reduction when selecting an embryo based on PRSs for multiple diseases. The figure is adapted from a published paper (Treff *et al.*, 2020a) and is based on the UK biobank sibling approach (Fig. 3C). For each sibling pair, one sibling is either selected at random (‘baseline’) or based on a weighted average of 11 PRSs (‘polygenic embryo screening’), as computed by a company offering the screen. For each disease, the figure shows the baseline risk (the proportion of randomly selected siblings who are affected) and the PES-based risk. The relative risk reduction is shown on top of each bar. The inset zooms in on the same data for the eight rightmost conditions depicted in the main panel (same order).

A study by Lencz *et al.* (2021) estimated the risk reduction following PES using both the liability threshold model (Fig. 3A) and the embryo genome simulation approach (Fig. 3B). Both modelling and simulations for schizophrenia and Crohn’s disease suggested relative risk reductions close to 50% when selecting out of five embryos (Fig. 4A). However, the corresponding absolute risk reductions are very small (<0.5% points), given that these diseases are uncommon. The baseline risk can increase due to either higher disease prevalence, as for type 2 diabetes, or when the parents are affected or have high genetic risk. In these cases, the relative risk reductions were smaller (Fig. 4A) but absolute risk reductions substantially increased.

Lencz *et al.* (2021) also studied the alternative embryo selection strategy of only avoiding the transfer of very high-risk embryos (‘deselection’) (Lencz *et al.*, 2021). This strategy would resemble the manner in which PRSs are being applied in adult clinical research: targeting the subset of individuals deemed ‘high-risk’ on the basis of extreme values of a PRS. It would also resemble current PGT-M and PGT-A practices. However, excluding embryos with risk at the top PRS percentiles (e.g. top 2% or 5%) and selecting at random among the remaining embryos was predicted to result in very little risk reduction. This outcome is a result of the low sensitivity of PRSs: while high PRS individuals are more likely to be affected, the vast majority of cases do not have a high PRS (Wald and Old, 2019). Thus, as opposed to screening in the adult population, embryo screening/selection is only beneficial when targeting the lowest-risk embryos.

Two studies by Genomic Prediction evaluated the expected risk reduction when screening for multiple diseases using nearly 12 000 sibling pairs from the UK Biobank (Treff *et al.*, 2020a; Widen *et al.*, 2022). For each sibling, Treff *et al* computed PRSs for 11 diseases (the nine mentioned in the study of Turley *et al.* (2021), as well as basal cell carcinoma and heart attack), and assigned each individual a ‘health score’, computed as the expected difference in healthy lifespan compared to the general population. Selecting the sibling with the higher score resulted in simultaneous risk reduction across all diseases. The relative risk reductions ranged from near zero for melanoma to 47% for heart attack, with a median across diseases of 20% (Fig. 4B). Widen *et al* added Alzheimer’s disease, atrial fibrillation, asthma, gout, inflammatory bowel disease, ischemic stroke, major depressive disorder, obesity, and schizophrenia. Selecting the sibling with the higher health score significantly reduced the risk for most diseases, with the rest not significantly differing from zero. The median relative risk reduction across diseases was ∼8%, and the maximum was again achieved for heart attack (∼22%). However, given that some of the conditions are relatively mild or preventable, prospective parents may prefer to focus on just one or a few severe diseases rather than the entire list.

A white paper by Orchid Health (ORCHID GUIDES, 2023) used both the liability threshold model and the sibling pairs approach to obtain risk reduction estimates similar to those of published studies. For example, using the liability threshold model, the relative risk reduction when selecting against one of eight diseases was in the range 25–46% (selecting out of five embryos). Absolute risk reductions were high with a history of the disease in a first- or second-degree relative of the embryo. An online risk reduction calculator is available (https://portal.orchidhealth.com/risk-calculator).

#### Examples for the expected risk reduction when screening for a single disease

**Figure 5.**
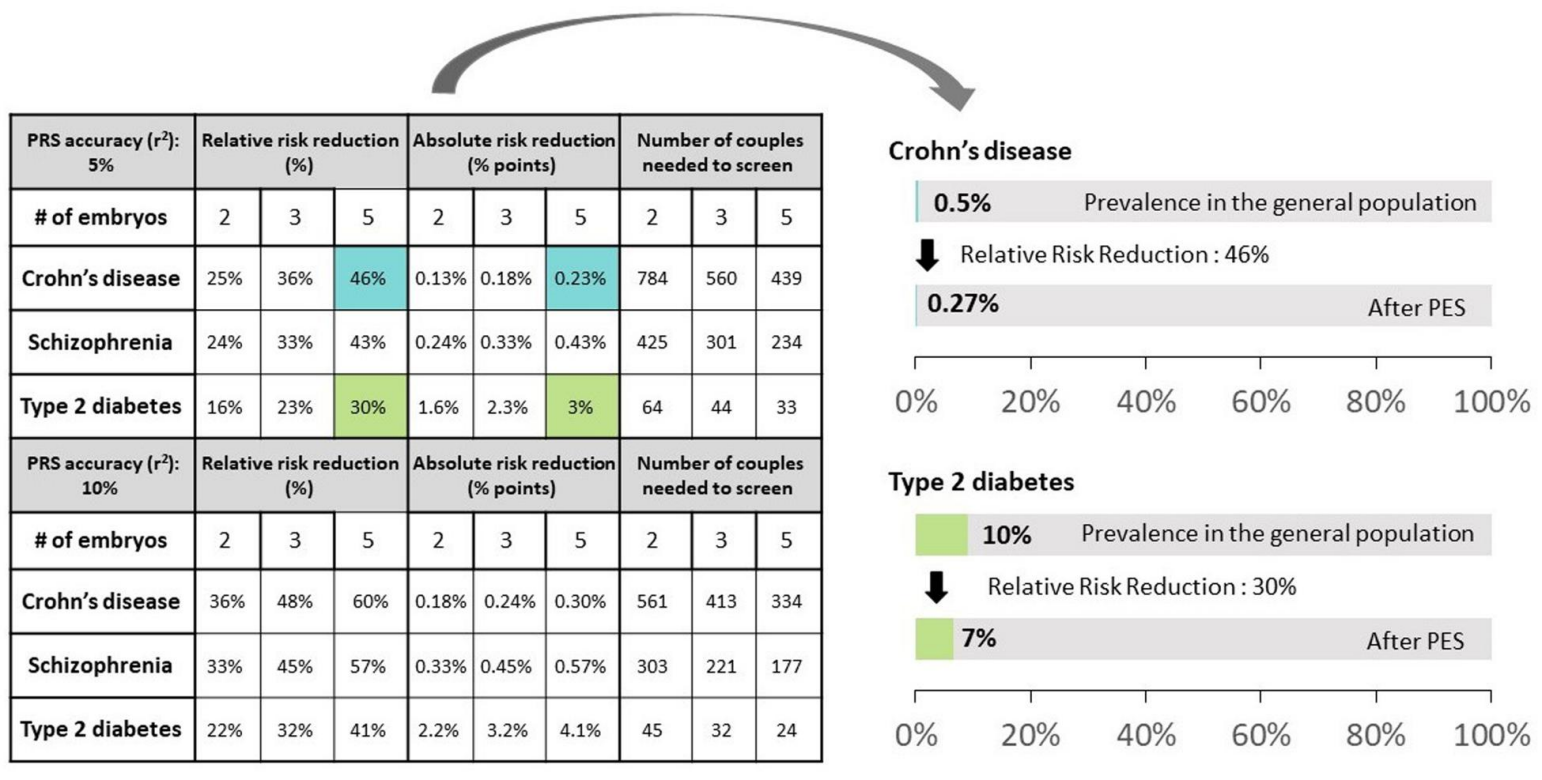
**Examples for the best-case-scenario expected risk reduction.** The table (left) uses a modelling approach (Lencz *et al.*, 2021), as implemented in the online risk reduction calculator (https://pgt-p-outcome-calculator.shinyapps.io/selectioncalc/). We assume that embryos from a single oocyte retrieval cycle were screened with a polygenic risk score (PRS) for a single disease and that the embryo with the lowest PRS was selected for transfer and was born. We present results for three diseases, whose prevalence is estimated as 0.5% for Crohn’s disease (GBD Inflammatory Bowel Disease Collaborators, 2020), 1% for schizophrenia (Perala *et al.*, 2007), and 10% for type 2 diabetes (Centers for Disease Control and Prevention). The accuracy of the PRS, as measured by r^2^ (the proportion of variance in liability explained by the PRS), was previously estimated at ≈6% for Crohn’s disease, ≈7% for schizophrenia (Lencz *et al.*, 2021), and ≈9% for type 2 diabetes (Ge *et al.*, 2022). We therefore show results for lower and upper bounds of *r*^2^ = 5% and 10%, respectively. The absolute risk reduction is the difference in risk between selecting an embryo at random (equal to the disease prevalence) and selecting based on PRS: absolute_risk_reduction= disease_prevalence—risk_of_selected_embryo. The relative risk reduction is the absolute risk reduction as a proportion of the initial risk: absolute_risk_reduction/disease_prevalence. The number of couples that would need to be screened in order to prevent a single case is computed as 1/absolute_risk_reduction. The bars on the right demonstrate the risk reduction for two diseases, Crohn’s disease and type 2 diabetes, when selecting the embryo with the lowest PRS out of five and when the PRS accuracy is *r*^2^ = 5%. For each disease, the upper bar demonstrates the risk of an embryo selected at random (equal to the population prevalence), while the lower bar shows the risk of a child born after PES.

Figure 5 provides examples of the expected outcomes of PES for a single disease based on the models of Lencz *et al.* (2021). We assume selection of the embryo with the lowest PRS for one of three chronic diseases with differing genetic architectures and prevalence: Crohn’s disease, schizophrenia, and type 2 diabetes. The figure reports the expected relative risk reduction, the absolute risk reduction, and the number of couples needed to screen to prevent a single case. Outcomes are shown when selecting out of two, three, or five embryos, all of whom are assumed to be born if transferred (see next section). We report results for two levels of PRS accuracy, providing approximate lower and upper bounds on the expected utility of PES for the listed diseases (Wray *et al.*, 2021).

Figure 5 shows that relative risk reductions increase rapidly with the number of embryos (see also Fig. 4A). However, the gains gradually diminish as the number of embryos increases beyond five (Fig. 4A) (Lencz *et al.*, 2021). Increasing the PRS accuracy also increases risk reduction. Absolute risk reductions are very small, particularly for the rarer diseases, leading to higher numbers needed to screen. Type 2 diabetes, which has the greatest prevalence, has the lowest relative risk reduction but higher absolute risk reduction and lower number needed to screen.

To permit researchers and clinicians to evaluate the expected (single-disease) risk reduction under various settings, we implemented the models of Lencz *et al.* (2021) in a web-based calculator (Lencz *et al.*, 2021) (https://pgt-p-outcome-calculator.shinyapps.io/selectioncalc/). The calculator, which is publicly and freely available, allows users to vary the number of available embryos, the PRS accuracy, the disease prevalence, the PRS percentile and disease status of the parents, and the selection strategy. The relative and absolute risk reductions can be plotted against most of these parameters.

### Factors that will limit the expected gains

In this section, we discuss modeling limitations and practical factors that may decrease the effectiveness of PES in reducing disease risk (Fig. 2).

####  

##### Low PRS predictive power

Risk reductions would be lower for diseases with a less predictive PRS. For some diseases, such as schizophrenia, Crohn’s disease, and type 2 diabetes (Figs 4A and 5), even the currently limited PRS accuracy can theoretically result in large relative risk reductions. For other conditions, such as anorexia (Watson *et al.*, 2019), amyotrophic lateral sclerosis (Restuadi *et al.*, 2022; Dou *et al.*, 2023), osteoarthritis (Lacaze *et al.*, 2022), chronic back pain (Tsepilov *et al.*, 2023), Tourette syndrome (Tsetsos *et al.*, 2023), abdominal aortic aneurysm (Hellwege *et al.*, 2023) and many cancers (Yang *et al.*, 2023), current PRS accuracy is smaller and risk reductions will consequently decrease.

##### Small absolute risk reductions

Even impressive relative risk reductions of up to 50% will usually translate to extremely small absolute risk reductions. For schizophrenia, with a prevalence of ∼1% (Perala *et al.*, 2007), a 50% risk reduction implies that the risk of the offspring decreases from 1% to 0.5%, i.e. an absolute risk reduction of 0.5% points. The probability of the child being unaffected would increase from 99% to 99.5%, and the number of couples that would need to perform PES to eliminate a single case would be 200. For Crohn’s disease, with a prevalence of ∼0.5% (GBD Inflammatory Bowel Disease CollaboStors, 2020), the absolute risk reduction would be merely 0.25% points even given a 50% relative risk reduction.

##### Modeling assumptions

The main approach for predicting risk reductions (Fig. 3A) relies on a popular, yet highly simplified, quantitative genetic model (Falconer, 1967). Further, published estimates ignore assortative mating, i.e. preferential mating between individuals with more similar phenotypes than would be expected by chance (Yengo *et al.*, 2018; Horwitz *et al.*, 2023). In the context of PES, the effective accuracy of a PRS will diminish proportionally to the correlation between the PRSs of parents (Young, 2023). However, the correlation is small for most diseases (Rawlik *et al.*, 2019; Sunde *et al.*, 2023). See Section 10 in the Appendix of Lencz *et al.* (2021) for an expanded discussion on the impact of modeling assumptions.

##### The transferred embryo may not be born

All three approaches for estimating risk reductions (Fig. 3) implicitly assume that the transferred embryo will be born. Thus, the ‘number of embryos’ parameter in these models more realistically corresponds to the number of live births per oocyte retrieval cycle. Further, these approaches do not specify whether, in case of implantation failure or miscarriage, the intended parents will initiate a second cycle or instead select the next best embryo. Similarly, current risk reduction estimates do not consider the case when parents select the next best embryo for their second child. Transferring an embryo other than the one top-ranked will lead to smaller risk reductions than predicted under the naïve models.

##### Maternal age or diminished ovarian reserve

The expected gains of PES decrease rapidly when there are fewer than five viable embryos per IVF cycle (Figs 4A and 5), ultimately reaching zero when only a single embryo is available. Therefore, PES may be much less beneficial for patients of advanced maternal age or for couples suffering from severe sub/infertility characterized by low treatment response and/or poor embryo development (Smith *et al.*, 2015).

##### Parents with non-European or admixed ancestry

PRS accuracy decreases when the population in which the PRS is applied has a different ancestry than the population in which it was developed (Scutari *et al.*, 2016; Ding *et al.*, 2023). Given that the largest genetic studies to date were conducted in populations of white Americans and white British (Sirugo *et al.*, 2019; Mills and Rahal, 2020), existing PRSs are less accurate in populations of Southern European, Middle-Eastern, South Asian, East Asian, and especially African ancestry. In African populations, accuracy reductions may exceed 75% (Martin *et al.*, 2019; Prive *et al.*, 2022; Kachuri *et al.*, 2024). The decreased accuracy will then diminish the expected gains of PES (Turley *et al.*, 2021). With the development of multiple non-European biobanks (Al Thani *et al.*, 2019; Ishigaki *et al.*, 2020; Wei *et al.*, 2021; Fatumo *et al.*, 2022; Walters *et al.*, 2023; Wong *et al.*, 2023; Ziyatdinov *et al.*, 2023), as well as methods for transferring PRSs across populations (Kachuri *et al.*, 2024), the magnitude of this problem may decline in the future.

In couples having admixed ancestry (e.g. African Americans or Latin Americans), there is an additional concern. Due to the randomness of Mendelian segregation, embryos may differ in their ancestry proportions. As many PRSs are correlated with ancestry, embryos with more (say) European ancestry may have consistently lower or higher risk scores (Curtis, 2018; Kim *et al.*, 2018; Haworth *et al.*, 2019; Conti *et al.*, 2021; Ding *et al.*, 2023). As a result, ranking embryos based on PRS may effectively become ranking based on ancestry. This would not only eliminate the potential health gains of PES, but is also socially undesirable. While PRSs can theoretically be adjusted for ancestry (Khera *et al.*, 2019; Thompson *et al.*, 2022), it is yet unclear whether all dependence can be removed (Zaidi and Mathieson, 2020).

##### Lower accuracy and relevance of current PRSs in future generations

A unique aspect of PES is the use of PRSs for adult diseases in individuals not yet born. Published risk reduction estimates assume that current PRSs, which were developed based on data from present-day adults, will sustain their accuracy decades into the future. However, this seems unlikely given evidence of variability in PRS accuracy across age groups and environments (Mostafavi *et al.*, 2020; Wang *et al.*, 2023). This problem is exacerbated given the already weak performance of PRSs in the adult screening setting (Hingorani *et al.*, 2023). The magnitude of the future decline in PRS accuracy is difficult to predict.

A related problem is that some of the diseases screened today may become less relevant for the adults of the next generation. For example, heart attack, which is a major cause of death today, may become more preventable (Oostveen *et al.*, 2023). Similarly, genetic factors that are associated with smoking have a major effect on the lifespan of present-day older adults. This is reflected in recent GWASs of lifespan (Joshi *et al.*, 2016) and health span (Jukarainen *et al.*, 2022), but is likely to become less relevant in the future.

##### Inability to validate PES experimentally

Given the many decades it will take to evaluate health outcomes, it is impossible to conduct controlled trials to demonstrate the effectiveness of PES (Grebe *et al.*, 2024). Consequently, medical providers may hesitate to offer a non-validated medical test. On the other hand, *BRCA* pathogenic variants are now screened as part of PGT-M in many countries despite the lack of corresponding trials (https://www.hfea.gov.uk/pgt-m-conditions/). However, an important difference is that *BRCA* variants have a known biological mechanism (Stoppa-Lyonnet, 2016).

#### Practical factors that will have at most a minor effect

##### Inaccuracy in computing the PRS based on an embryo biopsy

DNA testing in adults is usually based on blood or saliva samples, where DNA is abundant. In contrast, genetic testing of embryos is typically based on a trophectoderm biopsy of just 5–10 cells. The limited input material can lead to allele dropout and incomplete genome coverage, reducing the analytical validity of the screen (Ginod and Dahan, 2024; Grebe *et al.*, 2024). However, many methods are available to combine noisy embryo data with high-quality parental DNA data to generate accurate embryo genotypes. PES providers have demonstrated very high concordance of polygenic scores between single cell and bulk samples, including from born babies (Treff *et al.*, 2019b; Kumar *et al.*, 2022; Li *et al.*, 2024). The remaining limitations include the need (in some methods) to collect DNA from a close relative (in addition to the future parents) and the deficiency of current technologies in genotyping *de novo* and structural variants. While such variants are usually not included in PRSs, their inclusion in future PGT methods may improve risk prediction.

##### Reduced accuracy of polygenic scores within the family

Several studies demonstrated that PRSs can be substantially less accurate in predicting differences in outcomes between siblings compared to differences between unrelated individuals (Kong *et al.*, 2018; Selzam *et al.*, 2019; Okbay *et al.*, 2022). One reason is that PRSs are often correlated with ancestry and therefore represent not only genetic but also environmental exposures. Additionally, an apparent association between an allele and a phenotype may represent home environment factors correlated with the presence of the same allele in the parents (Young *et al.*, 2019). Given that sibling embryos would share the same ancestry and home environment, we expect weaker associations between alleles and phenotypes across the embryos, and thus lower gains of PES (Turley *et al.*, 2021). However, these phenomena mostly impact cognitive and behavioural traits, and the reduction in accuracy when predicting differences in disease status is much smaller (Lello *et al.*, 2020).

### Informational benefits

PES patients are provided with information on the genetic risk profile of their future children, regardless of which embryo is selected and regardless of whether the PRSs are even used for selection. Theoretically, early knowledge of a future child’s PRS profile could help parents prepare accordingly and better protect their child’s health (Tellier *et al.*, 2021). However, no data exists on such benefits. Further, any putative benefits would be limited by the probabilistic nature of PRSs (Lencz *et al.*, 2021; Turley *et al.*, 2021), the late age of onset for most diseases screened, the lack of specific preventive interventions for many diseases (Kumuthini *et al.*, 2022; Centers for Disease Control and Prevention, 2023; World Health Organization, 2023), and the low effectiveness of DNA-based risk communication in changing health behaviour (Marteau *et al.*, 2010; Hollands *et al.*, 2016; Driver *et al.*, 2023). Moreover, gathering predictive genomic information about a (future) minor is generally considered a violation of their right not to know, which is part of their informational autonomy, unless this serves a clear medical interest of the child (Borry *et al.*, 2009; Siermann *et al.*, 2024).

## Possible harms to PES patients, their offspring, and society

**Figure 6.**
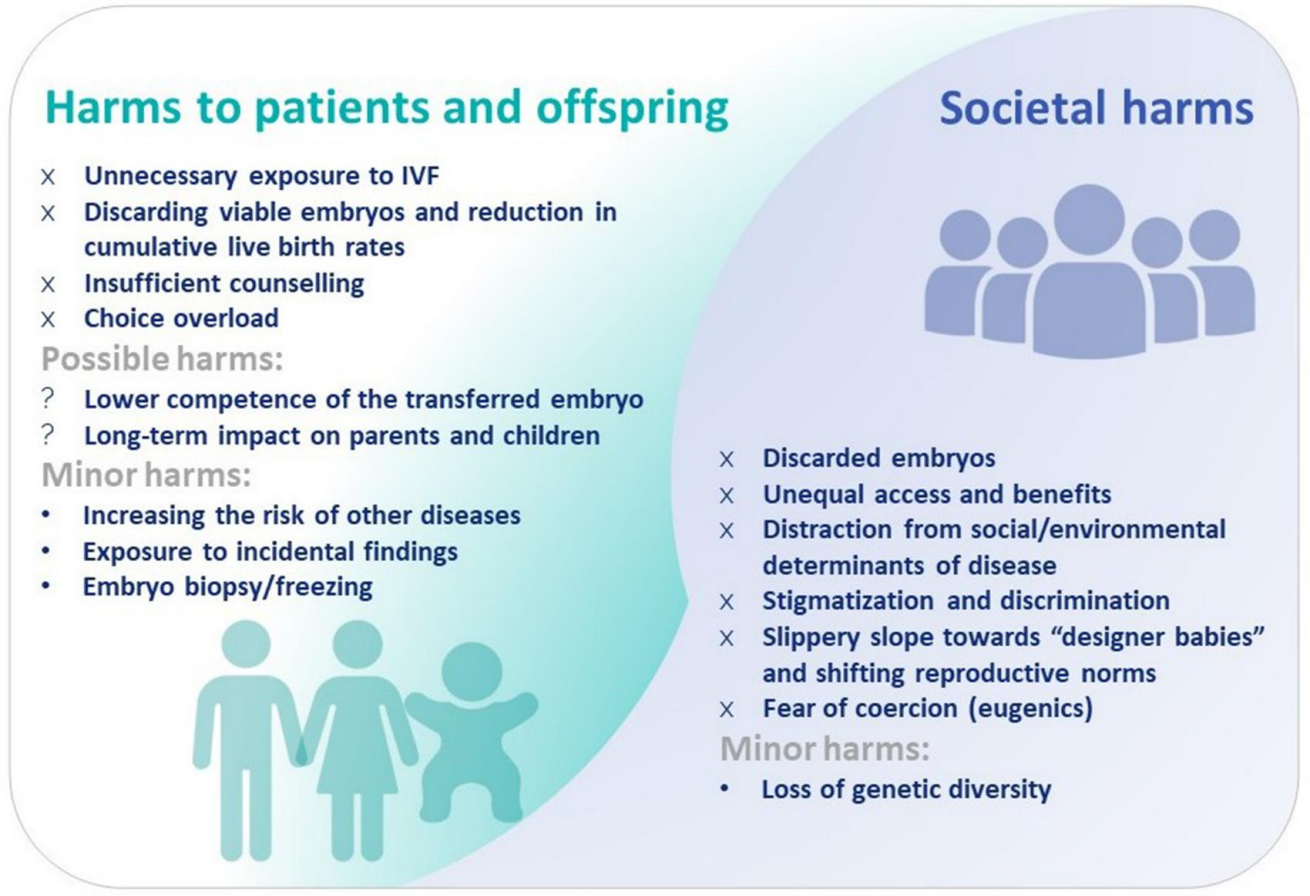
A visual summary of the possible harms to patients, future children and society due to PES.

In this section, we consider potential harms due to PES. We divide these harms according to who is affected: the patients and their future offspring, or society. We further highlight which harms are proven and which are currently speculative or expected to have a minor impact. The possible harms are summarized in Fig. 6.

### Possible harms to PES patients and their future offspring

####  

##### An unnecessary exposure to IVF treatment

The health gains of PES cannot materialize without IVF, and PES is currently primarily marketed only to IVF patients (https://www.lifeview.com). However, PES could theoretically be offered to all prospective parents, even in the absence of any medical indication. While IVF treatment is considered relatively safe, it is nevertheless associated with a number of risks and harms (Ginod and Dahan, 2023). Risks to the prospective mother include ovarian hyperstimulation, bleeding, infection, pain, injury, and pregnancy complications (ESHRE Clinic PI Working Group *et al.*, 2021). Risks to children conceived through IVF include low birth weight, preterm birth, and birth defects, although it is unclear whether these outcomes are related to the treatment or to the subfertility of the parents (Berntsen *et al.*, 2019). Increased mortality in the first year after birth has also been reported (Rodriguez-Wallberg *et al.*, 2020). Other harms include prolonged discomfort and stress, as well as IVF being a considerable expense.

##### Discarding healthy embryos and a reduction in cumulative live birth rates

In case the top-ranked embryo is transferred but not born, parents may choose to either use the next-best embryo (compromising on smaller risk reductions) or opt for another IVF cycle. In the latter case, all embryos other than the one transferred will have to be discarded or indefinitely stored. These embryos may be free of high-penetrance pathogenic variants, aneuploidy, or structural variants. Further, even embryos with above-average PRSs have a high likelihood of developing into adults free of all of the diseases screened (Hingorani *et al.*, 2023). Discarding such viable embryos will necessarily result in a reduction in cumulative live birth rates per IVF cycle.

##### Insufficient genetic counselling and market pressures

The probabilistic and correlational nature of polygenic scores makes them difficult to explain to non-experts. The terminology can be confusing and ambiguous: for example, risk can be presented as absolute (lifetime, age-based, or group-based), relative (to various reference points), or as a percentile (Lazaro-Munoz *et al.*, 2021; Turley *et al.*, 2021; Polyakov *et al.*, 2022; Grebe *et al.*, 2024). Moreover, most prospective parents will have little insight into the lived experiences of people with the conditions screened for, limiting the extent to which a well-considered judgement can be made.

The complexity of PES and the ambiguity in presenting its expected benefits leave room for market pressures. For example, PES could be marketed in either relative (‘50% risk reduction’) or absolute terms (‘0.25%’, for a disease with prevalence 1/200). Unless regulated, companies could choose to emphasize the more impressive relative risk reductions, leaving patients uninformed about the small absolute gains (Lencz *et al.*, 2021; Turley *et al.*, 2021) and about their actual risk. Similarly, companies may emphasize the best-case estimates, overlooking the many practical limitations (see above section) (Turley *et al.*, 2021; Forzano *et al.*, 2022; Polyakov *et al.*, 2022; Zappala *et al.*, 2023).

Given these considerations, independent and thorough genetic counselling, which informs patients on both quantitative and qualitative limitations, will be a prerequisite for informed decision-making (Grebe *et al.*, 2024). However, as a practical matter, this may turn out to be very difficult (Terek *et al.*, 2021; Forzano *et al.*, 2022; Johnston and Matthews, 2022; Siermann *et al.*, 2023). In case PES becomes widespread, shortages in the genetic counsellor’s workforce (Pereira *et al.*, 2022) might make it even more difficult for clinics to provide adequate counselling.

##### Choice overload

Ranking the embryos based on their risk profiles for 10–20 diseases might be overwhelming to patients (Lazaro-Munoz *et al.*, 2021). This is especially difficult given that no single embryo is expected to have a consistently low risk for all diseases screened. Choice overload is known to hamper autonomous decision-making, rather than enable it (Schwartz, 2004). Indeed, (Schwartz, 2015), a few prospective parents have elected not to transfer any embryo (Treff *et al.*, 2019a; Ray, 2021; Reynolds, 2022), providing anecdotal evidence to choice-induced paralysis (Schwartz, 2015) during PES. It was argued that the challenge of choice overload also affects other critical life decisions, such as career choice (Anomaly, 2023). However, the fact that a problem exists in one setting should not justify introducing it in another, particularly given the potential for discarding viable embryos and performing unindicated IVF cycles.

A potential solution to circumvent choice overload is to offer patients a ranking of the embryos based on an overall ‘health score’. For example, one company ranks embryos based on the predicted increase in healthy lifespan compared to the population mean (Treff *et al.*, 2020a). While this may reduce choice overload, parents may still prefer to examine the risk of individual diseases or even use third-party services to examine the risk for polygenic diseases or traits not screened, as anecdotally observed (Bloomberg News, 2022). Additionally, parents may prefer to weigh the screened conditions differently. For example, while Genomic Prediction’s ‘health score’ is dominated by prevalent conditions such as heart attack, parents may wish to more strongly avoid other conditions, such as relatively uncommon diseases that are notable in their family history, early-onset diseases, or psychiatric disorders. The only study of choice overload in the context of PES is a survey of eight PES patients by Genomic Prediction, revealing that 6/8 reported no or low regret and low anxiety and 7/8 found the embryo report easy to understand (Reynolds, 2022).

#### Possible harms on which data is lacking

##### Lower competence of the first transferred embryo

In the absence of pathogenic single-nucleotide or structural variants, embryos are currently prioritized for transfer based on their expected live birth potential. This is evaluated using their visible morphology during development, sometimes with the support of time-lapse monitoring (Pribenszky *et al.*, 2017; Armstrong *et al.*, 2019; Sciorio and Meseguer, 2021). With PES, the embryo with the most favourable PRS profile may not necessarily be the one recommended for transfer based on morphology, which may reduce implantation, pregnancy, and live birth rates. Anecdotal data from a conference abstract suggested no impact of PES on IVF success rates (Eccles *et al.*, 2022), but no published studies currently exist.

##### Negative impact of the knowledge of the child’s risk profile

PES provides parents with data on the genetic risk profiles of their embryos. The impact of this information on long-term health-related behaviour of parents and children is unknown and might be negative. When selecting an embryo with low risk of given diseases, parents may perceive their children as ‘immune’ and maintain a less healthy environment or refrain from seeking care (Ahn and Lebowitz, 2018; Turnwald *et al.*, 2019; Furrer *et al.*, 2023; Grebe *et al.*, 2024). This might cancel out any gains achieved by PES. Similarly, children informed of their own low genetic risk may adopt unhealthy habits. Further, knowing that one’s genetic profile has been ‘optimized’ may have unpredictable and adverse psychological impacts.

Given the limited pool of embryos to select from, the embryo transferred will almost always have a genetic risk for at least some of the diseases screened that is higher than the population average (https://gist.github.com/scarmi/3835f1ca0a9c77d61dbdcf8d85c91daf). Access to this information may lead to either desirable or undesirable degrees of watchful monitoring and (over)interpretation of symptoms, a phenomenon labelled as ‘healthy ill’ (Hubbard, 1993). Given the low positive predictive values of PRSs, there is an additional risk of overdiagnosis and overtreatment (Lewis and Green, 2021).

Several guidelines consider the predictive genetic testing of children for late-onset conditions to be at odds with their right to an ‘open future’, unless there are effective treatments or opportunities for prevention during childhood (European Society of Human Genetics., 2009; Hercher, 2020). Some, however, consider an ‘open future’ not to be a right of the child, but rather one interest of the child to be weighed against other potential interests, including good health (Garrett *et al.*, 2019). The relevant right may be better framed and specified as the right to informational self-determination, more specifically the right not to know (de Wert, 1992). In the context of PES, this may be particularly relevant for stigmatizing, difficult-to-treat, psychiatric conditions.

#### Minor possible harms

##### Exposure to incidental findings

The genome sequence of the prospective parents will become known during PES, either by direct sequencing during PGT or implicitly based on their embryos’ genomes. This may lead to unexpected discoveries of pathogenic variants (whether or not actionable) (Poli *et al.*, 2019; Griffin and Gordon, 2023), such as variants predisposing parents to breast cancer, aortic aneurysm, or Alzheimer’s disease (Vears and Amor, 2022). No data is available on the policies of PES companies regarding incidental findings. While the discovery of pathogenic variants can be lifesaving, it can also be a source of immense stress (Houdayer *et al.*, 2019).

##### Increasing the risk of a disease not screened

It is well known that many pairs of complex diseases and traits are genetically correlated; namely, variants that are associated with one condition are also associated with the other (Watanabe *et al.*, 2019). This phenomenon is known as pleiotropy. In the context of PES, a possible concern is inversely correlated conditions, where the same variants are associated with decreased risk for one condition but increased risk for another. If two inversely correlated conditions are screened, it would be rare to find an embryo with low genetic risk for both. If only one disease is screened, then PES may inadvertently increase the risk of the unscreened disease. The extent of this risk has been quantified (Lencz *et al.*, 2021; Turley *et al.*, 2021). The risk of pleiotropy is most clearly evident in livestock, where breeding farm animals for a single trait (e.g. egg laying in fowl or milk production in dairy cattle) has impaired the welfare of the animals (Muir and Craig, 1998; Rauw *et al.*, 1998; Millman *et al.*, 2000; Oltenacu and Algers, 2005).

Despite these initial concerns, inspection of the actual genetic correlations between pairs of human polygenic diseases suggests that most correlations are either null or positive (Zheng *et al.*, 2017; Treff *et al.*, 2020a; Yong *et al.*, 2020; Lencz *et al.*, 2021 (Widen *et al.*, 2022). A positive correlation is expected to enhance the gains achieved via PES: selecting an embryo with a low risk for one disease will decrease the risk for the other, even if not screened. Examples of positively correlated diseases are type 2 diabetes and myocardial infarction (Goodarzi and Rotter, 2020), most pairs of psychiatric diseases (Lee *et al.*, 2021), most pairs of substance abuse disorders (Bogdan *et al.*, 2023), and the latter and inflammatory bowel disease (Di Narzo *et al.*, 2021). A study by a company offering PES reported null or positive correlations for nearly all pairs of diseases they screen for (Widen *et al.*, 2022). Further mitigating the risk, a recent study suggested that many apparent genetic correlations are explained by cross-trait assortative mating rather than a shared genetic aetiology (Border *et al.*, 2022). Examples of inversely correlated diseases are few and mostly involve psychiatric disorders, such as schizophrenia and obesity (Zheutlin *et al.*, 2019), and bipolar disorder and ‘negatively perceived’ cognitive traits such as low educational attainment and low creativity (Turley *et al.*, 2021). While the risk of pleiotropy therefore seems minimal, it should nevertheless be seriously considered in any specific screening setting.

##### Harm due to embryo biopsy

PES requires embryo biopsy and cryopreservation, and therefore, PES patients are also exposed to any harm associated with these interventions (Ginod and Dahan, 2023). Recent research suggests that trophectoderm biopsy does not affect live birth rates (Awadalla *et al.*, 2021; Tiegs *et al.*, 2021), although there may be an increase in the prevalence of preterm birth and pregnancy complications such as preeclampsia and hypertensive disorders (Zhang *et al.*, 2019; Li *et al.*, 2021; Makhijani *et al.*, 2021; Alteri *et al.*, 2023). With respect to the impact of cryopreservation, one recent study suggested that frozen-thawed embryo transfer is associated with an increased risk of cancer in the offspring (Sargisian *et al.*, 2022). Another study reported that embryo vitrification is associated with higher birthweight, but found no other significant differences between children born after vitrified or fresh embryo transfer (Belva *et al.*, 2023). The question of harm due to PGT is still a subject of ongoing research, and long-term follow-up data are not generally available (Natsuaki and Dimler, 2018; Alteri *et al.*, 2023). In patients undergoing PGT on indication (e.g. PGT-M or PGT-SR), PES does not incur these additional harms (see the section below on patient groups).

### Possible harms to society

####  

##### Discarded embryos

The expected increased number of discarded viable embryos due to PES would be an inherent wrong for those who attribute a high moral value to embryonic human life, such as Roman Catholics (Best *et al.*, 2019). For those who attribute a lower, but not insignificant, moral status to human embryos, discarding embryos would be a sign of disrespect for human life (Bruno *et al.*, 2016). Alternatives to discarding, such as donation or even sale (Klitzman and Sauer, 2015), may be ethically or practically undesirable, especially given the potential labelling of these embryos as ‘genetically inferior’.

##### Unequal access and benefits

With the skyrocketing costs of IVF and PGT, PES will only be available to the wealthy. Moreover, the lower PRS accuracy in non-European populations imply that most potential future benefits of PES will be limited to people of European ancestries. To the extent that PES would lead to disease risk reduction, these benefits would therefore not be equally distributed among all groups in society, but rather increase inequality and deepen health disparities. It may also stimulate reproductive tourism (Bayefsky, 2016). It has been argued that collective funding of PES would address this concern (e.g. Treff *et al.*, 2022). Hypothetically, public funding could even decrease inequality, given that the people who will gain the most will be those most susceptible to disease (Treff *et al.*, 2022; Griffin and Gordon, 2023). However, as long as the benefits of PES are contested, it would be at odds with the public interest and distributive justice to publicly fund PES (de Wert and Dondorp, 2023).

##### Distraction from environmental and social determinants of health and disease

Some authors have argued that the adoption of PES as a means for reducing disease prevalence ‘may further de-emphasize environmental and social determinants of common diseases, drawing public attention away from structural solutions to health and disability challenges and toward individual responsibility for managing disease risk’ (Johnston and Matthews, 2022), thereby increasing injustice (Treff *et al.*, 2022; Denbow and Spira, 2023). Similar arguments have been previously made in the context of prenatal testing against disabilities (Asch and Barlevy, 2012). These harms may be exacerbated by increasing trends of genetic ‘essentialism’ (Dar-Nimrod and Heine, 2011), or ‘geneticization’ (Ginod and Dahan, 2024), and the increasing uptake of genetic testing inside (Hipp *et al.*, 2022; European IVF Monitoring Consortium for the European Society of Human Reproduction Embryology *et al.*, 2023) and outside (National Academies of Sciences *et al.*, 2020) reproductive medicine.

##### Stigmatization, discrimination, and the lived experience of health conditions

People with conditions screened in PES may become subject to stigmatization and discrimination (Poli *et al.*, 2019; Lazaro-Munoz *et al.*, 2021; Forzano *et al.*, 2022). Fears of stigmatization, along with unfamiliarity and overestimation of the burdens of living with these conditions (particularly those more heterogenous) could push parents further towards PES (Lazaro-Munoz *et al.*, 2021), reinforcing already existing negative stereotyping. This is especially a problem for conditions that are not considered diseases or disabilities by (part of) the community who has them, such as autism (Kristin Bumiller, 2008; Lilley *et al.*, 2023). Similar concerns were previously raised by members of the disability rights community regarding prenatal testing (Berens and Wasserman, 2022; Robinson, 2023) and PGT-M (Soniewicka, 2015). The prospect of PES extends the scope of this risk, insofar as the diseases screened are relatively more common. Considerations of stigmatization and discrimination particularly pertain to psychiatric conditions, where recent research has shown that biological explanations have not reduced social exclusion (Lencz *et al.*, 2022). It was argued that reducing the risk of a disease does not necessarily imply disrespect to people affected with the disease (Munday and Savulescu, 2021). However, others have argued that although the intention of screening may not be disrespectful, this does not prevent a stigmatizing effect in society or tangible emotional harm for disabled people (Boardman and Thomas, 2023).

##### Slippery slope towards trait selection

Although companies currently offering PES only screen for medical conditions, several critics fear that the introduction of PES will lead to screening for nonmedical or desirable traits, in the pursuit of ‘designer babies’ (Karavani *et al.*, 2019; Pagnaer *et al.*, 2021; Turley *et al.*, 2021; Kamenova and Haidar, 2022; Siermann *et al.*, 2023; Ginod and Dahan, 2024). In fact, PES for height and intelligence was originally offered by one company (MIT Technology Review, 2017). A similar slippery slope has been recently documented for elective sex selection (Bedrick *et al.*, 2022). The genome-wide data generated in PES could permit screening, for example, for cognitive and behavioral traits (Karavani *et al.*, 2019; Turley *et al.*, 2021), pigmentation phenotypes, and even overall ancestry. However some authors defend screening for traits in case it improves the overall well-being of the future child (Savulescu, 2001; Munday and Savulescu, 2021).

##### Fears of eugenics

Concerns about the spectre of eugenics have been raised since the advent of PGT (Robertson, 1992). These concerns have been amplified in the context of PES, given its broader scope, which covers late onset and chronic conditions, and its potential use for ‘enhancement’ (Karavani *et al.*, 2019; Lazaro-Munoz *et al.*, 2021; Turley *et al.*, 2021; Lencz *et al.*, 2022; Polyakov *et al.*, 2022). Eugenics movements have resulted in genocide, forced sterilization, and other interferences in reproductive choices during the first half of the 20th century. Critical features of eugenics that have led to these atrocities included government sponsorship, coercive/involuntary administration, restriction of procreative options for some groups, and a societally-focused goal of ‘improvement of stock’. It has been argued that with contemporary biomedical reproductive technologies, the aforementioned elements of eugenics are inverted, focusing now on parent-driven, voluntary decisions designed to enhance procreative options and the health of future offspring (Veit *et al.*, 2021; Treff *et al.*, 2022). By this view, PGT, including PES, would represent a more acceptable liberal eugenics (Agar, 1998; Anomaly, 2018). While this review is not designed to settle this debate, it is important to maintain clarity on the conceptual components of eugenics relevant to PES.

Regardless of the debate, the rank-ordering of future offspring on the basis of genetic qualities (Iltis, 2016), particularly when extended to traits and non-life-threatening conditions, might support an ideology legitimizing value judgements based on one’s genetic makeup (Mertes and Pennings, 2020; Lazaro-Munoz *et al.*, 2021). Moreover, individual choices collectively have a societal impact (Dive and Newson, 2022). Therefore, widespread adoption of PES may shape norms regarding reproductive choices, eventually reducing parents’ autonomy.

#### Minor possible harms

##### Loss of genetic diversity

One concern regarding population-scale implementation of PES is that it might lead to future loss of genetic diversity, due to the selection against alleles increasing disease risk or associated with undesired traits (Munday and Savulescu, 2021; Polyakov *et al.*, 2022; Ginod and Dahan, 2024). However, at least for the foreseeable future, the impact of selection is likely to be very small. This is because complex traits and diseases are influenced by hundreds or thousands of alleles (O'Connor *et al.*, 2019; Visscher *et al.*, 2021). In a single generation, PES can modify the mean polygenic score by at most ∼1SD (Karavani *et al.*, 2019). The latter is equal, due to principles of binomial sampling, to approximately the square root of the number of alleles associated with the disease or trait (Hsu, 2014). Thus, for example, if a disease is associated with 400 alleles, then even population-wide application of PES will reduce the average PRS by <5%. Therefore, it should take many generations for the population to experience a substantial loss of diversity. The expected change in the mean of traits is also expected to be very small (Karavani *et al.*, 2019). However, more formal estimates are not yet available.

## Possible issues with clinical implementation

### Where is PES legal and/or available?

PGT is not regulated in the USA, which is now home to at least three companies offering PES services. The company with the longest experience in the field, Genomic Prediction, self-reported in 2021 working with ‘approximately 200 IVF clinics on six continents’ (Genomeweb.com. 22 December 2021). The current cost of PES is ∼$1000 per embryo in Genomic Prediction (Breaking Latest News, 2023) and $2500 in Orchid, which also offers screening of rare pathogenic variants (Boston Globe, 2023).

The regulation of PGT and PES in seven European countries has been reviewed by (Siermann *et al.*, 2024). In the UK and the Netherlands, the use of PGT is limited to specific conditions, as determined by national regulatory bodies such as the UK’s Human Fertilisation and Embryology Authority (HFEA; https://www.hfea.gov.uk/pgt-m-conditions/). In France and Germany, PGT is approved by central authorities on a case-by-case basis. Under these two regulatory models, PES is unlikely to be permitted. In Belgium, Italy, and Spain, PGT is regulated by individual clinics, which leaves open the possibility of implementing PES, at least for medical conditions. However, the current disapproval of PES by clinicians and professional societies (see below) suggests that implementation of PES in the near future is unlikely even in these countries.

In other countries, PES is prohibited in Israel (https://www.health.gov.il/hozer/mr29_2013.pdf) and might be permissible in South Africa (Soni and Savulescu, 2021) and Singapore (Chin *et al*., 2024). No information is available regarding China; however, a recent report of a baby born following PES for diabetes (Wu *et al.*, 2023) suggests that PES is permitted in at least some cases. While information is lacking on the status of PES in many individual countries, a review of normative documents on PGT-M concluded that ‘the ethical acceptability of PGT-P seems limited’. However, it was also found that consensus is lacking and that views depend on local contexts (Siermann *et al.*, 2022).

### Who would use PES?

The benefits and harms of PES could vary considerably across patients (Grebe *et al.*, 2024). We identify three major groups of potential users of this technology (Table 2).

**Table 2.** A suggested ‘taxonomy’ of future PES patient groups, including key possible benefits and harms in each group.

No.	Group	Goal of IVF	Subgroup	Best-case relative risk reduction	Possible harms			
					IVF without indication	Biopsy/freezing	Choice overload	Trait selection
1	People requiring IVF	Have children	Sub/infertility	Small (few embryos)	Less likely	Yes	Minimal	Possible
			PGT			No		
			Gamete recipients	Possibly large, but small in absolute terms		Yes	Yes	
2	People with severe polygenic disease history	Reduce transmission of a specific disease	Prospective parents affected	Possibly large, even in absolute terms	Yes	Yes	Minimal	Unlikely
			Child affected					
			Other relatives affected	Intermediate				
3	Healthy people not requiring IVF	Interest in ‘healthier’ children		Possibly large, but small in absolute terms	Yes	Yes	Yes	Possible

The best-case risk reduction estimates are under a strategy of transferring only the embryo with the lowest genetic risk for one or more diseases.

The first group consists of people who already require IVF for any reason. It can be further divided into three subgroups: patients suffering from sub/infertility, patients requiring PGT (e.g. due to carrying a monogenic disease variant), and patients using gamete donation (including same-sex couples and single parents), in case they require IVF. In this group, PES will not be the reason for undergoing IVF treatments, and it is thus likely to be more acceptable. In addition, the PGT subgroup already requires embryo cryopreservation and biopsy, making PES even less harmful. However, it was argued that even for this type of ‘opportunistic screening’, benefits should be balanced with risks prior to implementation (de Wert *et al.*, 2021). Patients requiring IVF will tend to have fewer available embryos, either due to subfertility or due to embryos being found to have a pathogenic variant, and this will reduce the gains due to PES. In case the transferred embryo is not born, opting for additional IVF cycles rather than transferring already existing embryos will lower the chances of patients reaching their primary aim of live birth.

In the second group, one or both prospective parents and/or one or more of their close relatives (including an already-born child), suffer from a severe polygenic chronic disease. Examples include type 1 diabetes, schizophrenia, or asthma. These patients will consider PES for reducing the risk of their future children having a particular disease. PES is expected to be more acceptable in this group, as (i) relative risk reductions could be large, given the (assumed) normal fertility of the prospective parents; (ii) absolute risk reductions could also be large, given the high baseline risk; (iii) the decision on which embryo to transfer is based on a single PRS, which is expected to lead to little choice overload, and (iv) there is a lower risk of using PES in this setting for selection for traits. On the other hand, patients in this group do not require IVF for reproduction, and providing PES in this group would go beyond the presently accepted indications for PGT.

The third group of potential patients consists of healthy individuals who would pursue IVF and PES in order to improve the health of their future children. This group also includes gamete recipients who do not require IVF. PES in this group has the greatest number of potential disadvantages, given (i) the harms of IVF treatment, (ii) the cost of IVF treatment and the clinical resources it would consume; (iii) the lack of a specific medical problem; (iv) that children of these couples are expected to be in good health regardless of PES; (v) that these patients are the most likely to suffer from choice overload; and (vi) that they are more likely to screen for traits. On the other hand, patients in this group are expected to have relatively many embryos, such that large relative risk reductions could be achieved across multiple diseases.

It is too early to predict which patient group, if any, will adopt PES. Many patients already requiring IVF (group 1; with the possible exception of the gamete recipient subgroup) suffer from infertility or subfertility, which implies very few viable embryos and little gains. Also, the remaining embryos in group 1 are more likely to be reserved for transfer failure or for future children, further reducing gains. On the other hand, in group 1, PES may become integrated into the embryo evaluation process (Johnston and Matthews, 2022). The demand for PES among healthy couples (group 3) so far seems low (Eccles *et al.*, 2022), possibly given the harms and costs of IVF treatment. Couples with a disease history (group 2) would still suffer from the harms of an unindicated IVF treatment. However, PES in this group might offer specifiable gains while avoiding harms such as choice overload. Therefore, further evaluation of the benefits, harms, and demand for PES in this group will be a priority. An open question is the extent to which the definition of ‘severe polygenic disease history’ will expand to include additional conditions and family members, eventually blurring the boundary between this group and that of healthy couples (group 3).

### What are the attitudes of IVF patients?

A few US-based studies reported relatively high levels of interest and acceptance of PES among IVF patients. A conference abstract by a company offering PES reported that out of 113 IVF patients (with an unknown breakdown across indications), 56% elected to perform PES (Eccles *et al.*, 2022). A thesis reported a survey of n = 8 IVF patients who have taken a PES test (Reynolds, 2022). Motivations for using PES included, in the following order of importance, transferring the healthiest embryo, provider recommendation, gaining information, helping research, avoiding a specific disease, and recommendation by family. A survey of 641 IVF patients (or their partners) from New England in 2018 and 2021 found that 80% accepted whole-genome sequencing of embryos for reducing adult-onset disease risk (Neuhausser *et al.*, 2023). In-depth interviews with 26 US-based IVF patients (mostly white, female, and highly educated) found that all patients perceived PES as potentially beneficial. Almost all patients (24/26) were interested in PES, at least conditionally, including for traits (7/26). However, patients also expressed various concerns, particularly regarding information overload, psychological harm, the uncertainty of the outcomes, costs, and harm to the embryo. Many of the perceived benefits were informational (‘informed reproductive decision-making’), including preparation, reassurance, and curiosity (Barlevy *et al.*, 2024).

### What are the attitudes of the public?

Studies of the general public also reported wide acceptance of PES, particularly for medical conditions. In a representative US sample (n = 1457), 68% thought that PES (for either IQ, skin tone, height, schizophrenia, or diabetes) should be permissible (Zhang *et al.*, 2021). In another representative US sample (n = 633), reported in a conference abstract, 53% thought that PES is ethical and 37% were neutral (Peyser *et al.*, 2022). A large survey of a representative US sample (n = 6823) inquired about respondents’ attitudes towards PES for educational attainment under the assumption that they were already undergoing IVF and that PES was free and safe. The study found that 38% of the respondents would use PES with a likelihood >50%. The proportion of respondents who had no moral objection to PES was 48%. Willingness to use PES increased for respondents of age under 35 or with a higher education (Meyer *et al.*, 2023). Finally, another US-representative survey (n = 1435) found that 72% approved PES and 77% would vote to permit PES. If already undergoing IVF, 82% expressed an interest in PES. Approval for embryo selection for health conditions was 72–77%, while for traits it was only 30-36%. The main concerns of respondents were false expectations, eugenics, and stigmatization (Furrer *et al.*, 2024).

A limitation of these studies is that the high approval rates may reflect *prima facie* opinions on the general idea of PES, rather than ‘all things considered’ judgements. Particularly, the lay public may not fully appreciate the difficulties associated with IVF and PGT, as well as the probabilistic nature and limitations of polygenic scores and embryo selection, which are difficult to communicate over a short survey. Indeed, a randomized experiment (Furrer *et al.*, 2024) suggested that once informed about concerns, public approval of PES becomes more ambivalent.

### Clinical considerations

#### Published considerations

Adoption of PES at scale would have a substantial impact on IVF practice. Four studies explored the attitudes of healthcare professionals towards PES. These studies raised many concerns, both about the implementation of PES (from either the clinic side or the patient experience), as well as about its clinical utility and validity.

One study reported initial concerns raised by reproductive endocrinologists and infertility specialists, ethicists, social scientists, lawyers, geneticists, and genetic counsellors (Pereira *et al.*, 2022). The first concern was the expansion in IVF volume in case PES becomes widely adopted by healthy individuals (group 3). Particularly, this would exacerbate the already complex problems of discarding or indefinitely storing embryos. The second concern was that physicians may feel obliged to offer PES despite their own skepticism, if patient demand increases due to marketing pressures, if physicians feel pressure to use the newest technology despite a lack of clear advantage (‘the technological imperative’), or for avoiding liability due to not offering the test. The third concern was about disagreement with patients over the implementation of PES, including which conditions should be screened and which embryo should be transferred. The fourth concern was how to manage the expected increase in required counselling by physicians, genetic counsellors, and psychologists.

The second study interviewed 31 healthcare professionals (24 from Europe, 7 from North America; 16 working in genetics, 11 in reproductive medicine, and 4 in counselling) (Siermann *et al.*, 2023). The study identified several recurring clinical concerns: limited validity and utility, the non-diagnostic nature of PRSs, limited choices and difficulty in selecting embryos, discarding healthy embryos, and problems in understanding and explaining PES (Siermann *et al.*, 2023). Key social and ethical concerns included a slippery slope towards screening for traits, medicalization and commercialization of reproduction, stigmatization and discrimination, and violation of children’s autonomy (Siermann *et al.*, 2024).

The third study interviewed 27 reproductive endocrinology and infertility specialists from across the USA. Only 3/27 clinicians were willing to offer or discuss PES with their patients unconditionally, while 14/27 were willing to do so under specific circumstances (e.g. as part of the research, if initiated by the patient, or given a family history of a disease). Only 7/27 considered PES to be beneficial for selection purposes, and no clinician has expressed positive attitudes towards screening for traits. The main concerns regarding PES for health conditions were psychological harms, costs, not having enough embryos and discarding healthy embryos, the multifactorial, uncertain, and not-yet-studied nature of the outcomes, and the difficulty of governing PES (Barlevy *et al.*, 2024).

Finally, a conference abstract reported interviews with 11 US genetic counsellors. The concerns that were raised included ‘limitations in accessibility, lack of patient and provider education, lack in current research, and eugenics’. (Terek *et al.*, 2021).

#### Other considerations

Embryo ranking may not be robust across clinics and time, even for a given ranking strategy. At any given time, the instability of PRSs across studies (Ding *et al.*, 2022; Schultz *et al.*, 2022) and the large number of PRS-generating methods (Wang *et al.*, 2022) will result in clinics differing in their ranking of any given set of embryos. Additionally, as GWASs increase in size, PRSs improve in accuracy, raising the possibility of a periodic re-ranking of the embryos, similarly to the way variant pathogenicity annotations are updated (Macklin *et al.*, 2018; Mersch *et al.*, 2018; Carrieri *et al.*, 2019; Sharo *et al.*, 2023). For a realistic increase in the proportion of variance explained by a PRS over a few years from 5% to 10% (Lam *et al.*, 2019; Trubetskoy *et al.*, 2022), we expect the originally top-ranked embryo (out of five) to remain top-ranked only 54% of time (https://gist.github.com/scarmi/c8bcaca2aebb0b8886a301b4bdab84ab). Ranking inconsistencies and re-ranking might lead to frustration and confusion among patients (Mundt *et al.*, 2017; Tsai *et al.*, 2019; Wedd *et al.*, 2023), particularly when a re-ranked embryo has already been born.

Additional clinical considerations are also expected. First, the list of conditions to be screened will have to be decided by each clinic. Testing labs are expected to limit PES to severe/serious conditions, as has been suggested for prenatal screening (Bayefsky and Berkman, 2022) and preconception carrier screening (Dondorp *et al.*, 2015; de Wert *et al.*, 2021). Attempts to define the seriousness of a condition will need to take into account its lived experiences and overcome the lack of a widely agreed framework for judgments on disease severity (Kleiderman *et al.*, 2019; Kalsi, 2020). Second, clinics will need to develop policies regarding sharing the genomic data with the patients, who may then use third-party services to evaluate the embryos for other conditions, or even traits (Bloomberg News, 2022). Third, clinics will have to determine the relative importance of pathogenic variants in predisposition genes and a PRS for the same polygenic disease. While some clinics may always deselect carrier embryos, other clinics may combine the two sources of information into a single risk prediction model (Lee *et al.*, 2019; Kumar *et al.*, 2022) (High-Level Expert Group on AI, 2019). Finally, in countries where embryo discarding is not allowed, or whenever the prospective parents transfer no embryo (e.g. due to choice overload), PES may result in an increase in the number of embryos left in cryo-rooms for long-term storage.

#### Heterogeneity of the clinical considerations

Some of the clinical concerns may vary across patient groups (Table 3). For example, in people requiring IVF for reasons other than PES (group 1), there will be an increase in IVF volume only if parents opt for another cycle instead of using already existing embryos (e.g. after transfer failure). The number of people with severe polygenic disease history (group 2) is likely to be small, particularly under a strict definition of severity. In contrast, the pool of healthy people (group 3) is very large. However, PES demand in this group is unclear. In terms of discarding embryos, group 1 patients are less likely to discard embryos, as their primary goal in IVF is having children. In contrast, fertile patients of groups 2 and 3 will perform IVF only for reducing future disease risk, suggesting that they will be less interested in transferring embryos that were not top-ranked.

**Table 3.** Some clinical considerations that may vary across hypothetical PES patient groups.

No.	Group	Subgroup	Expected increase in IVF volume	Expected increase in discarded embryos
1	People already requiring IVF	Sub/infertility, PGT	Limited	Limited
		Gamete recipients		Probably limited
2	People with severe polygenic disease history		Probably small	Potentially large
3	Healthy people not requiring IVF		Possibly large, but demand unclear	

## Ethical considerations

The introduction of PES in the clinic has sparked widespread ethical concerns. In this section, we will look at PES through the lens of the widely accepted criteria for screening tests of Wilson and Jungner (Wilson and Jungner, 1968), as refined by others (e.g. Botkin, 2009). These criteria can be condensed to three principles: proportionality, respect for autonomy, and justice (de Wert and Dondorp, 2023).

### Proportionality (beneficence and non-maleficence)

The principle of proportionality requires evidence that the expected benefits of any intervention outweigh the possible risks. Arguments for and against PES therefore focus on the benefits and harms or, more generally, the well-being of prospective parents, future children, and the society (Griffin and Gordon, 2023). As we mentioned in the previous section (Tables 2 and 3), these benefits and harms should always be considered in the context of the specific patient situation (contextualized proportionality (Dondorp and de Wert, 2019)). The main predicted benefit of PES, as argued by the PES company Genomic Prediction, is a reduction in future disease burden, which would benefit both families and societies (Treff, 2020; Treff *et al.*, 2020a,b, 2022; Tellier *et al.*, 2021). For those patients who already require IVF, the company’s slogan ‘choice over chance’ implies the key argument that current IVF treatment already involves embryo ranking and selection, albeit without taking all available information into account (Griffin and Gordon, 2023). However, as mentioned in previous sections, actual clinical utility may be hampered by several practical limitations. Given the lack of decisive clinical validity data and the long list of possible harms described in previous sections, the benefits of PES do not clearly outweigh the harms (Karavani *et al.*, 2019; Turley *et al.*, 2021; Forzano *et al.*, 2022; Polyakov *et al.*, 2022; Siermann *et al.*, 2023).

Genomic Prediction also proposed that PES could contribute to personalized preventive medicine for future children identified as being at high risk for the screened disorders (Tellier *et al.*, 2021). This is consistent with a report that patients perceive PES as having informational benefits, such as preparing for the birth of the child (Barlevy *et al.*, 2024), and it may also be consistent with the principle of autonomy (see next section). However, as we described above, there is currently no evidence for the utility of this information, and the availability of the information might even be psychologically harmful to parents and children.

Another prominent argument supporting PES is based on ‘procreative beneficence’, holding that ‘couples (or single reproducers) should select the child, of the possible children they could have, who is expected to have the best life, or at least as good a life as the others, based on the relevant, available information’ (Savulescu, 2001; Treff *et al.*, 2022). In the context of PES, Munday and Savulescu pleaded for the adoption of a welfarist model, which restricts embryo selection to cases when it would clearly improve the well-being of the future child (Munday and Savulescu, 2021). They argued that this model is preferred over a disease model, which restricts PES to prespecified conditions, or a libertarian model, where PES could be used for any purpose. Based on the welfarist model, to the extent that PES is expected to improve well-being by reducing disease risk, it could be permitted. The main argument against this model is that we should not assume a direct causal link between disease risk and quality of life (Binkley *et al.*, 2022). Additionally, harms associated with PES may end up, even when balanced against the benefits, reducing well-being.

### Respect for autonomy

One of the main arguments for PES follows from the principle of respect for reproductive autonomy, suggesting that it is ultimately up to the parents to decide whether the benefit-risk balance is satisfactory from their point of view (Handyside, 2010; Tellier *et al.*, 2021; Treff *et al.*, 2022; Anomaly, 2023). In particular, patient autonomy may dictate that parents should be provided with any information about their embryos that they may be interested in. However, respect for autonomy should not simply mean that patients should be given any treatment they request. Rather, it entails guidance towards joint decision-making based on scientific insights (contributed by the health care provider, in this case preferably a genetic counsellor) and patient values, which need to be explored and made explicit in ‘interpretative’ pre-test counselling (Emanuel and Emanuel, 1992). In the case of PES, as we have elaborated in previous sections, informed decision-making would be very difficult, given the high level of complexity and the ease with which statistics can be misinterpreted (Hens *et al.*, 2013; Lazaro-Munoz *et al.*, 2021; Turley *et al.*, 2021; Forzano *et al.*, 2022; Johnston and Matthews, 2022; Polyakov *et al.*, 2022; Siermann *et al.*, 2023). This underscores the importance of training genetic counsellors to deal with the nuances of PES.

The empirically observed gap between the attitudes of clinicians and patients (Barlevy *et al.*, 2024) further complicates the interpretation of the principle of respect for autonomy. Should PES be offered to interested patients despite the reservations of clinicians (Pereira *et al.*, 2022)? Some researchers argue that the reproductive autonomy principle dictates that the test should nevertheless be offered (Treff *et al.*, 2022). Others may argue that given the possible harms of PES in some patient groups, particularly choice overload, it is the responsibility of clinicians not to offer the test (Dondorp and de Wert, 2011; Suter, 2018). Finally, the autonomy of future children to decide for themselves about predictive genetic testing, especially for untreatable and stigmatizing conditions, should be respected.

### Justice

The principle of justice raises several serious concerns regarding PES, as mentioned above. These include (i) unequal access (in case PES is deemed proportional) to people without means; (ii) unequal benefits to people with non-European ancestries; (iii) possible reinforcement of stigmatization and discrimination; (iv) possible diversion of healthcare resources (particularly IVF and genetic testing capacity) from more urgent needs, leading to infractions against distributive justice; and (v) a disproportional focus on individual responsibility, as opposed to structural injustices and social and environmental determinants of disease (Johnston and Matthews, 2022; Treff *et al.*, 2022), turning attention and resources away from these causes. All of these issues will increase injustice and thus argue against the introduction of PES.

### Policy statements

Several professional societies have issued statements regarding PES, including the ISPG (ISPG, 2021; Lencz *et al.*, 2022), ACMG (Abu-El-Haija *et al.*, 2023; Grebe *et al.*, 2024), ESHG (European Society of Human Genetics., 2021; Forzano *et al.*, 2022), and ESHRE (ESHRE, 2022), as well as the UK’s HFEA (Genomics in Education Programme., 2022). Given the wide spectrum of possible harms, as we have just surveyed, these statements were in consensus that PES is not yet ready for clinical use and that further research and societal debate is needed. Similar views have been expressed by other authors (Polyakov *et al.*, 2022; Treff *et al.*, 2022; Griffin and Gordon, 2023). However, given accumulating evidence in support of potential benefits and the limited impact of certain harms (e.g. pleiotropy, biopsy), future guidelines may become more nuanced.

## Future research

The number of IVF patients who have already performed PES is small. However, there are reasons to believe that PES will become more widely adopted in the future: (i) PES is straightforward to implement for any PGT lab employing genome-wide methods; (ii) it is relatively affordable for patients already going through IVF and PGT; (iii) several studies have projected health benefits; and (iv) there is an apparent public acceptance and interest in PES. On the other hand, many concerns have been raised over limited utility and potential harms, both personal and societal. It is therefore important to continue the evaluation of this technology.

We identified the following key research areas where studies of PES are most urgently needed.

First, studies are necessary to more accurately predict the risk reductions that are expected from PES. Current estimates have limited accuracy due to statistical modeling assumptions and the lack of genomic and phenotypic data from large sibships. Future statistical models should include embryo selection based on multiple conditions and complex selection criteria, estimation of per-family risk reductions, modelling of realistic IVF pipelines, and consideration of phenotypes that are influenced by both common and rare variants. Evaluation of selection outcomes in real data should extend beyond the UK Biobank, particularly into non-European and admixed populations. Studies of PRS accuracy across generations would clarify whether PES is expected lead to risk reduction for late-onset conditions. Continued research of the benefits and limitations of PRS screening in adults will further clarify its utility in the preimplantation setting.

Second, studies are urgently needed to evaluate the correlation between morphology- and PRS-based embryo ranks (across conditions and ranking strategies) and how PRS-based prioritization impacts pregnancy and live birth rates.

Third, we need a more nuanced understanding of the attitudes and the decision-making process of clinicians and patients. For patients, key open questions are their reasons to opt in or out of PES, their understanding of the technology, the conditions they are interested in screening, and their reasoning about which embryos to transfer, store, or discard, and how many embryos to generate. It is also crucial to identify and implement interventions, such as the development and dissemination of educational materials, that would close the gap between the attitudes of clinicians and patients and would ensure informed decision-making.

Fourth, data is needed on long-term outcomes in PES patients and their offspring. Open questions include their post-test regret (either on performing PES or on the identity of the embryo selected), the psychological impact on children, the impact on health-related behaviour and care-seeking, and how these outcomes change over time. Very long-term follow-up could evaluate the realized risk reductions; however, this would be beyond the scope of near-term research.

Fifth, cost-benefit analyses are unavailable but are urgently needed, especially given the relatively large numbers needed to screen as indicated in Fig. 5. Such studies should determine whether PES would be cost-effective for patients as a function of their country, ancestry, healthcare system, patient group, diseases screened, and embryo prioritization strategy. Cost-effectiveness studies from the payer’s perspective could inform healthcare providers and regulators on whether PES should be permitted, or even supported and reimbursed, and, if so, for which patient groups and diseases.

Finally, these statistical and epidemiological studies will require a further reflection by the bioethics community on whether PES should be permitted and/or provided, and, if so, on what conditions. Given that PES may be just another step in the direction of truly ‘comprehensive’ PGT (de Wert and Dondorp, 2023), a wider, anticipatory ethics agenda is important. Would such complete ‘rationalization’ of human reproduction be desirable?

## Conclusions

Screening human IVF embryos for the risk of future polygenic diseases is technically feasible and is already available to US-based patients. Statistical models, simulations, and real sibling outcomes (Fig. 3) suggest that PES could achieve large relative risk reductions in future children, at least under the best-case theoretical scenario (Figs 4 and 5). Despite the promising theoretical gains, multiple factors will limit the practical benefits (Fig. 2). The key issues include small absolute risk reductions, a limited number of embryos with live birth potential, low PRS accuracy in non-Europeans, and no guarantees on PRS accuracy and relevance in the distant future. Reduced PRS accuracy within the family and in embryo biopsies is expected to have a more minor impact.

Any proposed application of PES must consider the principle of proportionality and thus evaluate whether the benefits outweigh the potential harms. Our critical insight here is that proportionality must be evaluated in the context of the specific clinical scenario (Tables 2 and 3). We proposed to classify future patients into three groups: patients already requiring IVF (with or without PGT); individuals affected by or having a family history of a severe polygenic disease; and healthy individuals interested in the best health for their future children. The benefits, limitations, risks, and harms could differ widely between the groups.

The main harms to patients (Fig. 6) are the physical, emotional, and financial costs of IVF treatment (in case it is not otherwise indicated) and a possible reduction in IVF success rates. Choice overload, patient confusion, insufficient genetic counselling, and market pressures are also emerging as serious concerns. The long-term impact on parents and children is difficult to predict, but may also involve negative outcomes. Other harms, such as increasing the risk of another disease, risks due to the embryo biopsy, or exposure to incidental findings, seem minor.

Some of the risks of PES are societal in nature and are expected to increase injustice. One class of concerns include a slippery slope towards coercive eugenics, increasing demand for ‘designer babies’, and changes in reproductive values and norms, particularly when driven by market pressures. To achieve risk reductions, healthy embryos will have to be discarded, which might be a concern to some and is also a waste of healthcare resources. Importantly, the focus on genetic testing for reducing disease burden promotes views of genetic essentialism and distracts from environmental and social determinants of health. Ranking embryos by health conditions ignores the lived experience of people having these conditions. Additional concerns include stigmatization and discrimination against disease cases and an increase in health disparity. The main counterarguments include respect for reproductive autonomy (to the extent that a truly informed decision can be made) and that an embryo must be selected regardless of PES.

Given the current lack of clarity regarding the balance of benefits and harms, we recommend that PES is not yet introduced into the clinic. Nevertheless, given the potential theoretical benefits, it could be offered to patients within a research context and when supported by extensive patient counselling. We hope that our review will assist researchers, clinicians, regulators, and professional societies to develop empirically informed views and guidelines for this emerging technology.

## Data Availability

An online risk reduction calculator for a setting where embryos are prioritized based on their polygenic risk scores for a single disease appears at: https://pgt-p-outcome-calculator.shinyapps.io/selectioncalc/. Code and technical documentation for the calculator appear at: https://github.com/Lirazk/SelectionCalc.
